# Evaluating structural connectivity disruption after stroke: individual tractography or the use of a model-based approach?

**DOI:** 10.1016/j.nicl.2026.103967

**Published:** 2026-02-18

**Authors:** Franziska E. Hildesheim, Keith W. Jamison, Ilana R. Leppert, Anna Zumbansen, Anja Ophey, Thomas Funck, Amy Kuceyeski, Alexander Thiel, for the NORTHSTAR Study Group and the Alzheimer’s Disease Neuroimaging Initiative

**Affiliations:** a Lady Davis Institute for Medical Research, Montréal, QC, Canada; b Department of Neurology & Neurosurgery, McGill University, Montréal, QC, Canada; c Canadian Platform for Trials in Non-Invasive Brain Stimulation (CanStim), Montréal, QC, Canada; d Department of Radiology, Weill Cornell Medicine, New York, NY, USA; e Department of Computational Biology, Weill Cornell College, Ithaca, NY, USA; f McConnell Brain Imaging Centre, Montréal Neurological Institute, McGill University, Montréal, QC, Canada; g School of Rehabilitation Sciences, University of Ottawa, Ottawa, ON, Canada; h Music and Health Research Institute, University of Ottawa, Ottawa, ON, Canada; i Medical Psychology, Neuropsychology and Gender Studies, Center for Neuropsychological Diagnostics and Intervention, Faculty of Medicine and University Hospital Cologne, University of Cologne, Cologne, Germany; j Child Mind Institute, New York, NY, USA

**Keywords:** Stroke, Diffusion-weighted imaging, White matter, Structural connectivity, Tractography

## Abstract

**Background:**

Ischemic stroke disrupts structural connectivity between grey matter regions, influencing functional outcomes. Diffusion MRI-based tractography allows assessment of white matter damage and may guide rehabilitation. It remains unclear how structural connectivity disruption (SCD) estimated from individual tractography compares with model-based estimates using normative tractograms.

**Methods:**

Diffusion MRI data were analyzed from 23 individuals with subacute ischemic stroke and 60 healthy adults from two age-specific cohorts (Human Connectome Project/HCP, Alzheimer’s Disease Neuroimaging Initiative/ADNI3). In Experiment 1, SCD estimates from individual tractography were compared to scores from the model-based Network Modification (NeMo) tool in the stroke cohort. In Experiment 2, synthetic middle cerebral artery lesions were applied to healthy adults’ diffusion data to compare SCD estimates from tractography on synthetically lesioned versus complete data, to SCD estimates from the model-based NeMo tool. Rank-based linear mixed-effects models compared differences across approaches, cohorts, and 13 regions of interest (ROIs).

**Results:**

In stroke, SCD scores from individual tractography and NeMo were strongly correlated (R^2^ ≥ 0.81), with largest differences in the inferior parietal gyrus (median [IQR]: −4.2% [-9.2–1.4]). In healthy controls, NeMo underestimated SCD relative to individual tractography, with larger median SCD differences across ROIs in older (11.8% [7.7–13.3]) than younger adults (−6.7% [-9.3–-1.9]). Cohort and ROI explained 47–69% of variance in SCD difference across approaches.

**Conclusion:**

Both individual tractography and the model-based framework capture regional SCD patterns, with larger differences in older adults. The model-based framework is efficient for large-scale studies, while individual tractography may better capture patient- and age-specific SCD.

## Highlights

• Individual and model-based connectivity disruption estimates are strongly correlated.
• Disruption is underestimated by the model-based approach in older adults.
• Using age-specific tractogram reference sets may improve estimation accuracy.

## 1 Introduction

Ischemic stroke ranks among the primary causes of functional disability worldwide (Martin et al., 2025), often resulting in motor and cognitive impairments that compromise stroke survivors’ independence and quality of life (Gallucci et al., 2024, Einstad et al., 2021). These impairments vary extensively across individuals, with recovery trajectories and response to treatment showing considerable inter-individual variability (Martin et al., 2025, Cassidy and Cramer, 2017, Ramsey et al., 2017). Functional outcomes are influenced by a range of demographic factors including age (Osa Garcia et al., 2020, Johnson et al., 2022, Kim and Vemuganti, 2015), sex (Rexrode et al., 2022), and time since stroke (Johnson et al., 2022), as well as clinical variables such as initial functional status (Osa Garcia et al., 2020, Hildesheim et al., 2024), cognitive ability (Johnson et al., 2022), cardiovascular risk factors (Aam et al., 2021, Roth et al., 2023), receipt of reperfusion therapy (Menichelli et al., 2019, Crijnen et al., 2016), and genetic background (Kristinsson and Fridriksson, 2022, Balkaya and Cho, 2019). Structural brain characteristics, such as smaller lesion volumes, lower lesion load (Osa Garcia et al., 2020, Hillis et al., 2018, Feng et al., 2015), and better-preserved structural integrity of remote brain networks (Hildesheim et al., 2024, Salvalaggio et al., 2020, Bowren et al., 2022) have also been associated with more favorable recovery outcomes. A promising approach for improving stroke outcome prediction involves quantifying the extent to which specific grey matter (GM) regions are structurally disconnected from the broader brain network as a result of infarct damage. These disconnection measures can guide personalized rehabilitation planning tailored to the extent of brain damage and individual recovery potential (Thiel and Zumbansen, 2016).

Structural connectivity is commonly assessed using diffusion-weighted magnetic resonance imaging (DWI), which provides information about the direction and magnitude of water diffusion across brain tissues (Dmytriw et al., 2017). A widely used approach for modeling water diffusion is diffusion tensor imaging (DTI), which estimates a single dominant diffusion direction per voxel (Tae et al., 2018). Due to its use of a single-tensor model, DTI is limited in its ability to resolve complex fiber architectures, such as crossing or kissing fibers (Jeurissen et al., 2013, Auriat et al., 2015). To overcome this limitation, constrained spherical deconvolution (CSD) has been developed, which estimates fiber orientation distributions (FOD), allowing for the identification of multiple fiber populations within a single voxel (Jeurissen et al., 2013, Auriat et al., 2015, Jeurissen et al., 2014). By sampling from FODs, probabilistic tractography can reconstruct underlying white matter (WM) pathways, while accounting for complex fiber configurations (Behrens et al., 2007, Jeurissen et al., 2011). This approach allows to examine how stroke lesions affect the structural integrity of both local and remote GM regions.

In many clinical settings, diffusion-weighted imaging is not routinely included in the standard imaging protocol after stroke. To facilitate the quantification of structural connectivity disruption (SCD) in people without individual DWI data, model-based assessment tools have been developed (Kuceyeski et al., 2013, Griffis et al., 2021, Foulon et al., 2018). An example is the Network Modification (NeMo) tool, which estimates SCD by overlaying a subject’s lesion mask onto a normative tractogram reference set generated from diffusion data of 420 healthy adults (Kuceyeski et al., 2013). The tool quantifies connectivity disruption by identifying streamlines connecting a given GM region to all other atlas-defined GM regions and calculating the proportion of streamlines intersecting the infarct. The NeMo framework has been applied across a range of neurological conditions to investigate individual network disruptions, including stroke (Kuceyeski et al., 2014, Schaechter et al., 2023), and multiple sclerosis (Kuceyeski et al., 2015, Tozlu et al., 2021).

The extent to which SCD estimates derived from model-based approaches align with those obtained from individual tractography using subject-specific diffusion data remains unclear. We conducted two experiments to systematically compare these approaches. In Experiment 1, SCD scores estimated with the model-based NeMo framework (Approach 1) were compared with those derived from individual tractography in individuals with subacute ischemic stroke from the multi-center Non-Invasive Repeated Therapeutic Stimulation for Aphasia Recovery (NORTHSTAR) trial (Thiel et al., 2015). An important consideration in this comparison is that the NeMo framework relies on normative tractograms from healthy adults that are substantially younger than the stroke population and therefore may not accurately capture age-related structural differences in the brains of people with stroke. Moreover, individual tractography in people with stroke is based on diffusion data with missing information within the infarct, which may influence streamline reconstruction in the remaining brain network. Experiment 2 was designed to address these methodological differences using data from two age-specific healthy cohorts from the Human Connectome Project (HCP) Young Adult dataset (Van Essen et al., 2013) and the Alzheimer’s Disease Neuroimaging Initiative (ADNI3) database (Mueller et al., 2005). Synthetic lesions were applied to complete diffusion data, allowing SCD estimates derived from tractography on complete and synthetically lesioned data to be compared with each other and with estimates obtained from the NeMo framework.

## 2 Materials & methods

### 2.1 Data selection

**Fig. 1:**
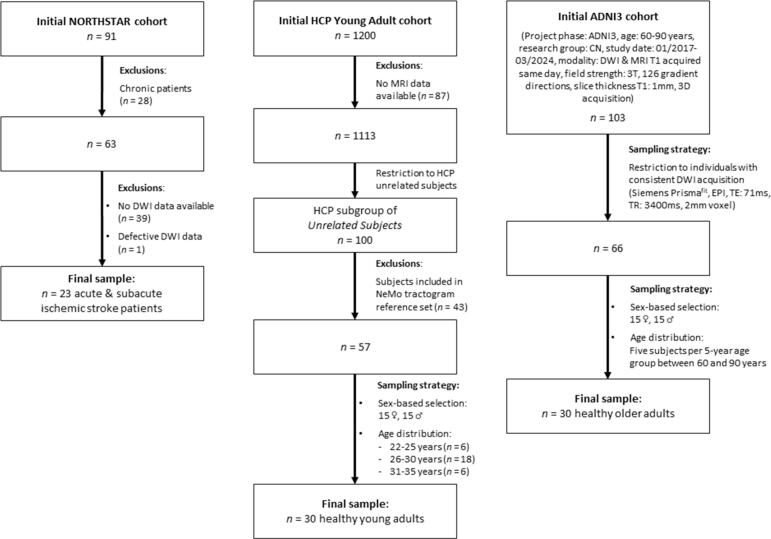
Data selection flowchart. NORTHSTAR: Non-Invasive Repeated Therapeutic Stimulation for Aphasia Recovery, HCP: Human Connectome Project, ADNI: Alzheimer’s Disease Neuroimaging Initiative, DWI: diffusion-weighted imaging, MRI: magnetic resonance imaging, NeMo: Network Modification, T: tesla, EPI: echo-planar imaging, TE: echo time, TR: repetition time, CN: individuals classified as cognitively unimpaired, ♀: female, ♂: male.

Three distinct datasets, comprising a total of 83 subjects, were analyzed in this study (see data selection flowchart in Fig. 1). For Experiment 1, 23 individuals with acute or subacute ischemic stroke from the prospective multi-center NORTHSTAR trial were included (Thiel et al., 2015). Individuals in the chronic phase were excluded, as progressing secondary neurodegeneration (Brunelli et al., 2023) and related changes in white matter over time (Goodman et al., 2023) can alter structural connectivity independently of the primary lesion, potentially confounding comparisons of SCD estimates across methods. Participants were recruited within 45 days of ischemic stroke onset in the left middle cerebral artery (MCA) territory and had a language score below age-adjusted norms in at least one of the three primary outcome measures, i.e., the Boston Naming Test (BNT) (Tombaugh and Hubley, 1997), the Token Test (TT) (De Renzi and Faglioni, 1978), and the Semantic Verbal Fluency Test (SVF) (Tombaugh, 1999). The study protocol was approved by the institutional ethics committee of each participating study center, and informed consent was obtained from all participants in accordance with the Declaration of Helsinki.

For Experiment 2, two cohorts of healthy adults were selected. The first cohort comprised 30 young healthy adults aged 22 – 35 years, drawn from the HCP-Young Adult (HCP-YA) database of the Washington University – University of Minnesota Consortium (https://www.humanconnectome.org/). The HCP-YA dataset includes neurologically healthy individuals who underwent extensive medical, neurological, and psychiatric screening (Van Essen et al., 2013). To ensure biological independence within the cohort, participants were selected from the predefined HCP-YA *100 Unrelated Subjects* subgroup, which is representative of the full HCP-YA sample with respect to age, sex, and MR image quality and contains no familial relationships. From this subgroup, participants were eligible if they were not included in the tractogram reference set used by the NeMo framework (Approach 1). The final HCP cohort consisted of 15 female and 15 male participants, selected according to the predefined HCP-YA age brackets, with six individuals aged 22 – 25 years, 18 individuals aged 26 – 30 years, and six individuals aged 31 – 35 years. This distribution was chosen to achieve a mean age comparable to that of the NeMo tractogram reference set (age range: 22 – 37 years, mean ± SD: 28.7 ± 3.7 years). Potential familial relationships between participants in the present cohort and those included in the NeMo tractogram reference set were not assessed.

The second cohort comprised 30 older healthy adults aged 60 – 90 years, selected from the ADNI3 database (adni.loni.usc.edu) (Mueller et al., 2005). Participants were drawn from the cognitively normal (CN) group and had no apparent memory impairment. Eligible participants were selected from studies conducted between January 2017 and March 2024 and were required to have both 3D T1-weighted structural magnetic resonance imaging (MRI) and DWI acquired on the same day. To minimize acquisition-related variability, only data acquired using identical DWI parameters were included (Siemens Prisma^fit^ scanner, echo-planar imaging, TE: 71 ms, TR: 3400 ms, isotropic voxel size = 2 mm, 126 gradient directions). The ADNI was launched in 2003 as a public–private partnership, led by Principal Investigator Michael W. Weiner, MD. The primary goal of ADNI has been to test whether serial MRI, positron emission tomography (PET), other biological markers, and clinical and neuropsychological assessment can be combined to measure the progression of mild cognitive impairment (MCI) and early Alzheimer's disease (AD). For up-to-date information, see www.adni-info.org.

### 2.2 Data acquisition

For Experiment 1, the NORTHSTAR data were acquired on 3 T (tesla) MRI scanners with varying manufacturers and scanner models across study centers. To ensure consistency, the harmonized ADNI2 protocol was employed. An anatomical T1-weighted image (1 mm isotropic resolution, TE: 2.98 ms, TI: 900 ms, TR: 2300 ms) was acquired, followed by a T2-weighted fluid attenuated inversion recovery (FLAIR) sequence (2 mm isotropic resolution, TE: 75 ms, TI: 2500 ms, TR: 9000 ms). Single-shell DWI data were acquired with either 32 (*n* = 8), or 64 (*n* = 15) diffusion directions (2 mm isotropic resolution, TE: 90 ms, TR: 10000 ms, *b* = 1000 s/mm^2^).

For Experiment 2, the HCP data of healthy controls were acquired on a Siemens Skyra 3 T scanner. Structural T1-weighted images were acquired with an isotropic resolution of 0.7 mm (TE: 2.14 ms, TI: 1000 ms, TR: 2400 ms). High-resolution multi-shell diffusion data with reverse phase-encoding direction were collected across six separate series using 90 gradient directions (1.25 mm isotropic resolution, TE: 89.5 ms, TR: 5520 ms, *b* = 1000, 2000, 3000 s/mm^2^). Structural and diffusion data were pre-processed using the HCP minimal pre-processing pipeline prior to download from the database (Glasser et al., 2013).

The ADNI3 data of healthy controls were acquired on 3 T Siemens Prisma^fit^ scanners and included a 3D T1-weighted MR scan (1 mm isotropic resolution, TE: 3 ms, TI: 900 ms, TR: 2300 ms), and multi-shell diffusion data with 126 gradient directions (2 mm isotropic resolution, TE: 71 ms, TR: 3400 ms, *b* = 500, 1000, 2000 s/mm^2^). For details on ADNI2 and ADNI3 MRI protocols, refer to the ADNI website (https://adni.loni.usc.edu/methods/documents/mri-protocols/).

### 2.3 Lesion Delineation & synthetic lesion mask generation

In Experiment 1, stroke lesions were manually delineated based on hyperintensities on each individual’s FLAIR image, which was linearly coregistered to the subject’s T1-weighted image. Corresponding hypointensities on the T1-weighted image were used to further refine lesion boundaries. Lesion masks were created independently by two experienced raters and subsequently verified by a third rater.

In Experiment 2, synthetic stroke lesion masks were generated using the Digital 3D Brain MRI Arterial Territories Atlas (Liu et al., 2023), which defines territories of arterial blood supply based on probability maps derived from lesion distributions in 1298 individuals with acute stroke. The frontal and parietal regions of the left MCA territory were extracted to create two binary lesion masks in Montréal Neurological Institute (MNI) space. These left-hemispheric synthetic lesions were selected to represent anterior and posterior MCA strokes, both of which are commonly associated with aphasia, thereby making them comparable to the lesion locations observed in individuals with stroke from the NORTHSTAR cohort. The frontal MCA lesion (MCAFL) and the parietal MCA lesion (MCAPL) had respective volumes of 149.84 cm^3^ and 97.67 cm^3^ in MNI standard space.

### 2.4 Assessment of structural connectivity disruption within the NeMo framework (Approach 1)

To compute structural connectivity disruption scores using the NeMo framework (Approach 1), each subject’s infarct mask was non-linearly registered to MNI152 space and uploaded to the NeMo online toolbox (v2.1a8) (Kuceyeski et al., 2013). Default parameters were modified to perform probabilistic tractography using iFOD2 (improved 2^nd^ order integration over fiber orientation distribution) with anatomical constraints to white matter (ACT, anatomical constrained tractography), streamline weighting via SIFT2 (Spherical-deconvolution Informed Filtering of Tractograms 2), 1 mm dilation of parcels from the Automated Anatomical Labeling (AAL116) atlas (Tzourio-Mazoyer et al., 2002), and exclusion of streamlines with both endpoints located within the same AAL116 parcel. The algorithm overlays the subject’s infarct mask onto a normative tractogram reference set derived from diffusion data of 420 healthy adults from the WU-Minn HCP Young Adult database, which were pre-processed using the HCP minimal pre-processing pipeline (Glasser et al., 2013).

The NeMo framework (Approach 1) calculates region-wise structural connectivity disruption scores, referred to as Change in Connectivity (ChaCo) scores by the tool’s developers, using the following formula (Kuceyeski et al., 2013): $\mathrm{ChaCo}=F_{L}/F_{T}$, where *F_T_* denotes the summed SIFT2 weight of all streamlines connecting a given ROI to all other GM regions of the atlas, and *F_L_* represents the summed SIFT2 weight of those ROI streamlines intersected by the lesion mask. ChaCo scores quantify the proportion of disrupted streamlines connecting to a given region and range from 0 to 1, where 1 indicates full structural disconnection of a GM region, and 0 indicates no disruption.

### 2.5 Estimation of structural connectivity disruption in individual tractography

**Fig. 2:**
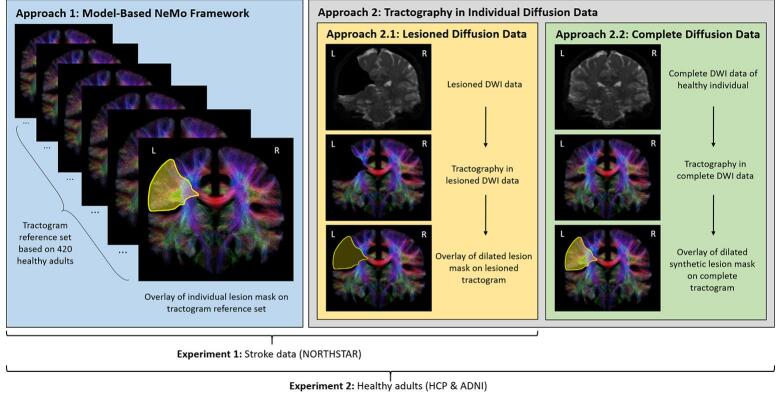
Experimental workflow for the estimation of structural connectivity disruption. Experiment 1: Comparison of SCD estimates derived from the model-based NeMo framework (Approach 1), and patient-specific individual tractography (Approach 2.1) in 23 individuals with subacute ischemic stroke. Experiment 2: Comparison of SCD estimates from complete individual diffusion data (Approach 2.2) in 30 young (HCP) and 30 older (ADNI) healthy adults, with SCD estimates from synthetically lesioned diffusion data of the same individuals (Approach 2.1) and the model-based NeMo framework (Approach 1). NORTHSTAR: Non-Invasive Repeated Therapeutic Stimulation for Aphasia Recovery, HCP: Human Connectome Project, ADNI: Alzheimer’s Disease Neuroimaging Initiative, NeMo: Network Modification, DWI: diffusion-weighted imaging, L: left, R: right.

In Experiment 1, SCD scores estimated using the NeMo framework (Approach 1) were compared to SCD scores derived from tractography performed on individual diffusion data (Approach 2.1) in people with stroke. In Experiment 2, SCD scores were estimated in healthy control subjects from the HCP and ADNI cohorts using two distinct approaches: a) tractography performed on pre-processed, complete diffusion data, with the synthetic lesion mask overlaid onto the resulting tractogram (Approach 2.2), and b) tractography performed on pre-processed diffusion data which had been synthetically lesioned by removing the diffusion signal within the synthetic infarct mask prior to tractography (Approach 2.1). These two approaches allow the isolation of methodological differences by comparing SCD estimates from the same diffusion data and identical lesion locations under controlled conditions. Note that Approach 2.1 refers generally to tractography performed in the presence of a lesion, whether it is a naturally occurring lesion in people with stroke (Experiment 1) or a synthetic lesion in healthy controls (Experiment 2). An overview of the comparison design and workflow is presented in Fig. 2.

#### 2.5.1 Pre-processing of structural & diffusion data

A detailed overview of all data processing steps and the software tools used is provided in Supplementary Fig. 1. Diffusion data from individuals with stroke and ADNI subjects were corrected for susceptibility-induced distortion using Synb0-DisCo v.3.0 (synthesized b0 for diffusion distortion correction), an algorithm that enables distortion correction for data without a reverse phase encoding sequence (Schilling et al., 2019, Schilling et al., 2020). After eddy current and motion correction (Andersson and Sotiropoulos, 2016, Jenkinson et al., 2012), the b0 image was brain-extracted. The pre-processed b0 image and the brain-extracted T1-weighted image (Fischl, 2012) were linearly coregistered (Jenkinson et al., 2002). The coregistered T1 image was then non-linearly registered to MNI152 space (Avants et al., 2008) to enable accurate mapping of the AAL116 atlas to individual diffusion space. For HCP subjects, structural and diffusion data were pre-processed using the HCP minimal pre-processing pipeline prior to data download (Glasser et al., 2013). The HCP pipeline included gradient nonlinearity correction, echo planar imaging distortion correction, eddy current and motion correction, and linear registration of the diffusion data to the subject’s T1-weighted structural image. In all three cohorts, the brain-extracted, unmasked T1-weighted image was segmented into GM, subcortical GM, WM, and cerebrospinal fluid (CSF) using MRtrix3 5ttgen (Smith et al., 2012, Dhollander et al., 2016). For the ADNI and HCP datasets, this segmentation was performed on the whole-brain T1 prior to the introduction of any synthetic lesions. The segmented T1 was then registered to diffusion space for ADNI and stroke data (already done for HCP data as part of the HCP pre-processing pipeline). For people with stroke and in the tractography approach in synthetically lesioned data (Approach 2.1), the lesion mask was incorporated as pathological tissue into the registered segmented T1 image prior to tractography, whereas for tractography on complete diffusion data in the HCP and ADNI dataset (Approach 2.2), the segmented T1 image was used without including the synthetic lesion, consistent with the NeMo framework (Approach 1). Minor corrections of the segmented T1 image were applied in some cases to address misclassification of WM hypointensities as GM due to insufficient intensity contrast. All data were visually inspected for quality and artifacts. Each processing step, including registration, atlas alignment, lesion masking, and tractography underwent thorough quality control for every subject across all three cohorts.

#### 2.5.2 Probabilistic tractography and extraction of region-specific tractograms

In both Experiment 1 and 2, tissue-specific response functions for WM, GM and CSF were estimated using the MRtrix3 implementation of the Dhollander algorithm to provide the basis for FOD reconstruction (Dhollander and Connell, 2016). Voxel-wise FODs were then estimated using CSD. For individuals after stroke, single-shell 3-tissue CSD (SS3T-CSD, MRtrix3Tissue) (Tournier et al., 2004) was applied, while multi-shell multi-tissue CSD (MSMT-CSD, MRtrix3) (Dhollander and Connell, 2016) was applied to the ADNI and HCP cohorts (Jeurissen et al., 2014, Tournier et al., 2010). WM tracts were reconstructed using probabilistic tractography (iFOD2, MRtrix3), anatomically constrained to WM (Smith et al., 2012, Smith et al., 2015). Tracking parameters included an FOD amplitude cutoff of 0.06, a maximum streamline length of 300 mm, and dynamic bidirectional seeding within the WM (tckgen seed_dynamic, MRtrix3), generating five million streamlines per subject. To improve biological plausibility and reduce biases related to streamline density, streamlines were weighted using SIFT2, with a fiber density threshold of 0.06 (Smith et al., 2015).

The AAL116 atlas was non-linearly registered to each subject’s native diffusion space, and parcels were dilated by 1 voxel to ensure inclusion of streamlines terminating at the boundary between WM and GM. Since the diffusion voxel size differed across cohorts, the dilation corresponded to 1.25 mm in HCP data and 2 mm in NORTHSTAR and ADNI data. A whole brain connectome matrix was constructed, assigning streamline endpoints to their corresponding AAL116 parcels in diffusion space, using a voxel-wise value lookup at each endpoint (Hickok and Poeppel, 2007). Thirteen left-hemispheric cortical regions of interest (ROI), pre-defined as core areas involved in speech and language processing and/or production, were selected for analysis based on established models of the language network and comprehensive reviews of language neuroanatomy (see Supplementary Table S1) (Price, 2012, Fridriksson et al., 2018, Fujii et al., 2016, Ardila et al., 2016, Bennett and Madden, 2014).

For each ROI, all streamlines connecting that region to all other GM regions of the AAL116 atlas were extracted to generate ROI-specific tractograms. Streamlines terminating outside the AAL116 atlas, i.e. in WM, as well as those with both endpoints located within the same AAL116 parcel, were excluded from the analysis. The subject-specific stroke lesion mask (Experiment 1) or the synthetic infarct mask (Experiment 2), both registered to diffusion space, were dilated by 1 mm and overlaid onto each ROI-specific tractogram to identify intersecting streamlines.

#### 2.5.3 Weighting of structural connectivity disruption scores in lesioned diffusion data

ROI-specific SCD scores for tractography derived from synthetically lesioned diffusion data (Approach 2.1) and from complete diffusion data (Approach 2.2) were calculated using the same formula as for ChaCo scores in the NeMo framework (Approach 1), namely $\mathrm{SCD}=F_{L}/F_{T}$ (Kuceyeski et al., 2013). To account for the potential direct overlap between an AAL116 atlas region and the infarct in tractography on lesioned diffusion data (Approach 2.1), the resulting SCD scores were weighted according to the following formula: ${\mathrm{SCD}}_{\mathrm{weighted}}=\left({(F}_{L}/F_{T}\right)*R_{\mathrm{NL}}+R_{L})/100$, where *F_T_* denotes the summed SIFT2 weight of all streamlines connecting a given ROI to all other GM regions of the atlas, *F_L_* represents the summed SIFT2 weight of those ROI streamlines intersected by the lesion mask, *R_NL_* represents the percentage of the dilated ROI not directly overlapping with the dilated infarct, and *R_L_* denotes the percentage of the ROI directly overlapping with the dilated infarct. *R_L_* was quantified by overlaying the dilated ROI with the dilated lesion mask in MNI space and calculating the proportion of the ROI’s volume that was directly infarcted. This weighted SCD score assumes that the portion of the ROI overlapping with the lesion (*R_L_*) is fully disconnected (i.e., SCD = 1).

An anatomically implausible overlap was observed after dilation for the combination of the synthetic frontal MCA lesion and the superior temporal pole (11%) and superior temporal gyrus (STG, 0.4%). Since the temporal pole and STG are not supplied by the MCA branches that give rise to the frontal MCA infarct, these overlaps are anatomically impossible. The intact portions of the ROIs were thus set to 100% to not overcorrect SCD scores for artificial overlaps. This adjustment was limited to these specific lesion–ROI combinations and did not affect any other SCD weightings.

The SCD weighting procedure was applied to the NORHSTAR dataset, as well as the synthetically lesioned data of the healthy control HCP and ADNI cohorts (Approach 2.1). For tractography derived from complete diffusion data (Approach 2.2), this additional SCD score weighting was not necessary.

### 2.6 Linear mixed-effects models to assess age effects in healthy controls

Statistical analyses were performed using RStudio (version 2024.12.1.563). Differences in SCD estimates were analyzed using rank-based linear mixed-effects models (LMM). These analyses were conducted only for Experiment 2, which used data from age-specific healthy cohorts to examine potential age-related differences in SCD across approaches. Separate LMMs were fitted for synthetic frontal and parietal MCA lesions to compare: a) the NeMo framework (Approach 1) versus individual tractography on synthetically lesioned diffusion data (Approach 2.1), b) individual tractography on complete (Approach 2.2) versus synthetically lesioned diffusion data (Approach 2.1), and c) the NeMo framework (Approach 1) versus individual tractography on complete diffusion data (Approach 2.2). In all LMMs, SCD difference scores were rank-transformed to account for non-normality and specified as the dependent variable. Fixed effects included the cohort of healthy controls (HCP, ADNI), the 13 ROIs, and the cohort x ROI interaction. Subjects were modeled with random intercepts to account for inter-individual variability. Estimated marginal means (EMMs) of the ranked SCD difference scores were computed for each cohort and ROI, and pairwise contrasts between HCP and ADNI within each ROI were derived to interpret the cohort x ROI interaction.

### 2.7 Correlation of SCD estimates with language outcomes in the stroke population

Functional language outcomes were assessed six weeks after baseline in the stroke population (NORTHSTAR). ROI-wise SCD scores derived from both the model-based NeMo framework (Approach 1) and individual tractography (Approach 2.1) were correlated with three language measures: Boston Naming Test, Token Test, and Semantic Verbal Fluency Test. Associations were quantified using R^2^, calculated separately for each ROI. This analysis was intended to provide an overview of relative association strength across methods rather than to develop a predictive clinical model.

### 2.8 Inter-individual variability and streamline distribution in healthy controls

Two exploratory analyses were performed to further characterize SCD estimates. First, inter-subject variability in SCD scores, referred to as ChaCo scores within the NeMo framework (Approach 1), was examined across the 420 individual tractograms in the NeMo reference set for the synthetic frontal and parietal MCA lesions introduced in Experiment 2. These individual ChaCo scores represent structural connectivity disruption estimates for each reference tractogram prior to averaging.

Second, seed density maps were generated for tractography on complete (Approach 2.2) and synthetically lesioned (Approach 2.1) diffusion data to examine the spatial distribution of streamline seeds used during probabilistic tractography. This exploratory analysis was performed on a few randomly selected cases from the healthy control HCP and ADNI cohorts.

### 2.9 Data Availability

Data supporting this study can be made available upon reasonable request to the corresponding author. The NeMo framework is an open-access online tool (https://kuceyeski-wcm-web.s3.us-east-1.amazonaws.com/upload.html) (Kuceyeski et al., 2013).

## 3 Results

### 3.1 Demographics and clinical data

**Fig. 3:**
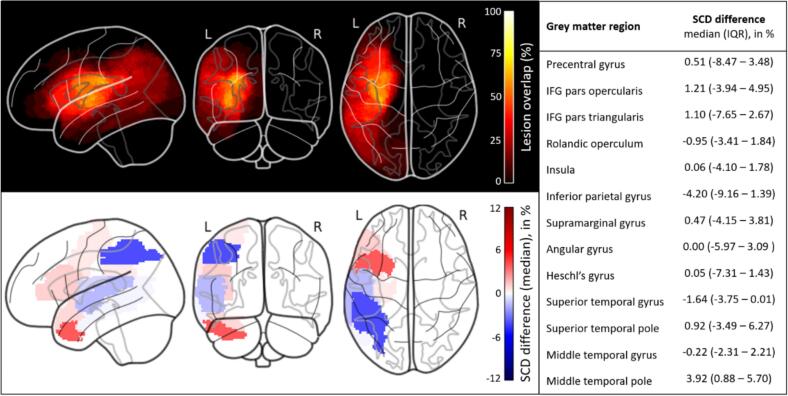
Lesion distribution and differences in structural connectivity disruption estimated with the model-based Network Modification tool versus individual tractography in people after stroke (Experiment 1). Top panel: Lesion distribution map of 23 individuals with stroke. Higher values indicate greater lesion overlap. Bottom panel: The glass brain visualization shows the median differences in structural connectivity disruption (SCD) scores across 13 grey matter regions from the AAL116 atlas. Blue indicates underestimation of SCD by the model-based NeMo framework (Approach 1) relative to individual tractography (Approach 2.1), whereas red indicates overestimation. Table: Median SCD difference scores with interquartile range. SCD: structural connectivity disruption, IFG: inferior frontal gyrus, AAL: Automated Anatomic Labeling, IQR: interquartile range, L: left, R: right.

Experiment 1 included 23 individuals with acute or subacute ischemic stroke, of which 52% were male (*n* = 12). The mean age at the time of stroke was 65.0 ± 10.0 years (range: 46 – 84 years). Participants were recruited 2 – 42 days after stroke (mean ± SD: 16.8 ± 11.1 days). The median normalized lesion volume was 63.8 cm^3^ (interquartile range/IQR: 20.1 – 84.5 cm^3^). A lesion distribution map is shown in Fig. 3. Stroke severity at initial assessment was mild to moderate, with a median National Institutes of Health Stroke Scale (NIHSS) score of 8.0 (IQR: 4.0 – 14.0). Common cardiovascular risk factors included hypertension (70%, *n* = 16), dyslipidemia (30%, *n* = 7), diabetes mellitus (26%, *n* = 6), and atrial fibrillation (22%, *n* = 5).

Experiment 2 included two groups of healthy adults. The younger cohort consisted of 30 healthy individuals (50% male, *n* = 15), aged 22 – 33 years (mean ± SD: 27.8 ± 2.9 years), selected from the WU-Minn HCP Young Adult dataset. These subjects were not part of the tractogram reference set within the NeMo framework (Approach 1). The older cohort included 30 healthy individuals (50% male, *n* = 15), aged 60 – 90 years (mean ± SD: 75.2 ± 8.3 years), selected from the ADNI3 database, with five subjects per five-year age bracket. Subjects in the ADNI group showed no evidence of cognitive impairment based on the Logical Memory II subscale of the Wechsler Memory Scale-Revised, the Mini-Mental State Examination, and the Clinical Dementia Rating.

### 3.2 Experiment 1: Differences in estimates of structural connectivity disruption between the NeMo framework and individual tractography in people with stroke

The Shapiro-Wilk test indicated non-normal distributions of SCD difference scores in several ROIs (*p* ≤ 0.05). SCD scores derived from the NeMo framework (Approach 1) and individual tractography (Approach 2.1) were highly correlated within each individual with stroke (*R*^2^ = 0.81 – 0.99; Supplementary Fig. 2). The NeMo framework (Approach 1) resulted in lower median SCD scores in four ROIs and higher SCD scores in nine ROIs relative to individual tractography (Approach 2.1). Greatest SCD differences were seen in the inferior parietal gyrus (median [IQR]: −4.2% [-9.2 – 1.4]), and the middle temporal pole (median [IQR]: 3.9% [-0.9 – 5.7]; Fig. 3).

### 3.3 Experiment 2: Differences in estimates of structural connectivity disruption in synthetically lesioned data in healthy controls

The Shapiro-Wilk test indicated non-normal distributions of SCD difference scores in several ROIs across both healthy control cohorts and synthetic lesion types (*p* ≤ 0.05).

#### 3.3.1 Comparison 1: NeMo framework (Approach 1) versus individual tractography on synthetically lesioned data (Approach 2.1) in healthy controls

**Fig. 4:**
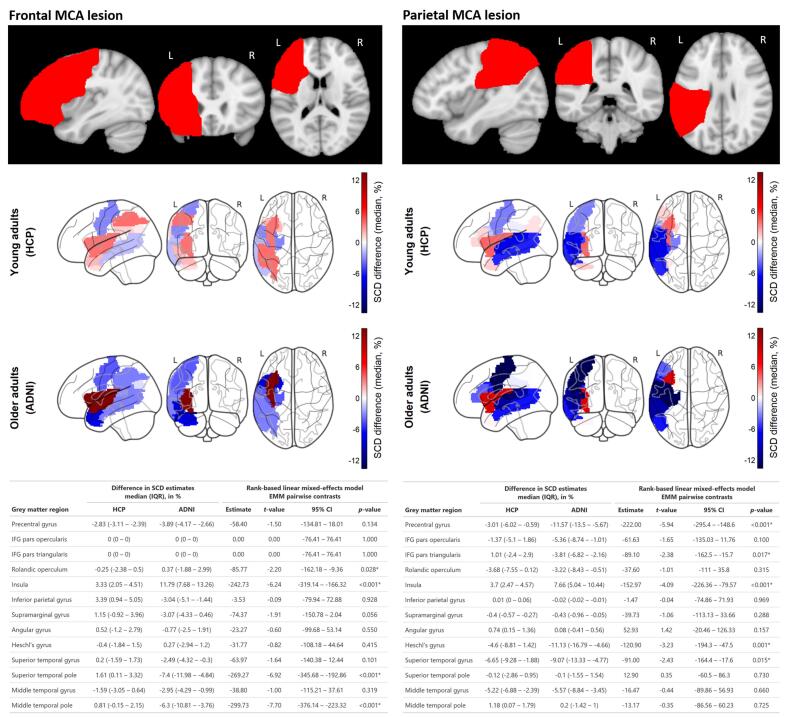
Differences in structural connectivity disruption scores estimated with the model-based Network Modification tool versus individual tractography on synthetically lesioned diffusion data of healthy adults (Experiment 2, Approach 1 vs. Approach 2.1). Top panel: Synthetic lesion masks in frontal and parietal middle cerebral artery territories. Middle panels: Glass brain visualizations show median differences in SCD scores across 13 grey matter regions from the AAL116 atlas for young and older adults. Blue indicates underestimation by the model-based NeMo framework (Approach 1) relative to individual tractography on synthetically lesioned data (Approach 2.1), whereas red indicates overestimation. Tables: Results from rank-based linear mixed effects models, with cohort and regions of interest as fixed effects and subject as a random intercept. Estimated marginal means of ranked SCD differences were calculated for each cohort and ROI, with pairwise contrasts between young and older adults. Statistical significance was defined as *p* ≤ 0.05 (*). HCP: Human Connectome Project, ADNI: Alzheimer’s Disease Neuroimaging Initiative, MCA: middle cerebral artery, SCD: structural connectivity disruption, IFG: inferior frontal gyrus, AAL: Automated Anatomic Labeling, ROI: region of interest, EMM: estimated marginal means, IQR: interquartile range, L: left, R: right.

In the first analysis, SCD estimates derived from the NeMo framework (Approach 1) were compared to SCD scores from tractography on synthetically lesioned diffusion data (Approach 2.1) in healthy control subjects. In young adults, median SCD differences between approaches remained below 5% across most ROIs, with the exception of the STG (median [IQR]: −6.7% [-9.3 – −1.9]) and the middle temporal gyrus (median [IQR]: −5.2% [-6.9 – −2.4]) for the parietal MCA lesion (Fig. 4).

In older adults, the NeMo framework (Approach 1) underestimated SCD scores relative to individual tractography (Approach 2.1) for all ROIs across both synthetic lesions, except the insula, for which the NeMo framework overestimated SCD scores. The largest median SCD differences in older healthy adults were observed in the insula (median [IQR]: 11.8% [7.7 – 13.3]) for the frontal MCA lesion and the precentral gyrus (median [IQR]: −11.6% [-13.5 – −5.7]) for the parietal MCA lesion.

Rank-based LMMs confirmed that older adults exhibited significantly larger SCD differences between approaches compared to young adults in four ROIs for the frontal MCA lesion and five ROIs for the parietal MCA lesion. Cohort and ROI accounted for 59% of the variance in SCD differences between approaches in the parietal MCA lesion and 56% of the variance in the frontal MCA lesion. Subjects explained an additional 2% and 1% of the variance, respectively.

#### 3.3.2 Comparison 2: Individual tractography on complete (Approach 2.2) versus synthetically lesioned data (Approach 2.1) in healthy controls

The second analysis compared SCD estimates derived from individual tractography on complete diffusion data (Approach 2.2) with scores obtained from tractography on synthetically lesioned data (Approach 2.1) in healthy controls. In young adults, median SCD differences between approaches remained below 3.5% across ROIs and infarct locations, whereas for older adults SCD differences were below 5.6%, with the exception of the insula, which again showed larger differences for both synthetic lesions (Supplementary Fig. 3).

Rank-based linear LMMs revealed significantly larger SCD differences between approaches in older compared to young adults in two ROIs for the frontal MCA lesion and five ROIs for the parietal MCA lesion. Cohort and ROI explained 69% and 67% of the variance in SCD differences between approaches in the parietal and frontal MCA lesion, respectively. Subjects explained an additional 1% of variance for both lesions.

#### 3.3.3 Comparison 3: NeMo framework (Approach 1) versus individual tractography on complete data (Approach 2.2) in healthy controls

The third analysis compared differences in SCD estimates derived from the NeMo framework (Approach 1) with SCD scores obtained with individual tractography on complete diffusion data (Approach 2.2) in healthy controls. Consistent with the first analysis, the NeMo framework (Approach 1) tended to underestimate SCD scores relative to individual tractography (Approach 2.2) in older adults. In young adults, median SCD differences between approaches remained below 3.1% across ROIs, except for the insula in the frontal MCA lesion, which again showed larger discrepancies (median [IQR]: 5.6% [3.9 – 6.8]). In older adults, SCD differences between approaches were larger, reaching up to −8.9% (IQR: −11.3 – −3.4) in the precentral gyrus.

Rank-based LMMs revealed significantly greater SCD differences between approaches in older compared to young adults in four ROIs in the frontal and parietal MCA lesion, respectively (Supplementary Fig. 4). Cohort and ROI explained 53% and 47% of the variance in SCD differences between approaches in the frontal and parietal MCA lesion, respectively, with subjects accounting for an additional 2% of the variance in the parietal MCA lesion.

### 3.4 Inter-individual variability of SCD estimates within the tractogram reference set of the NeMo framework

Exploratory analyses revealed considerable inter-individual variability in SCD scores across the 420 healthy individuals comprising the tractogram reference set of the NeMo framework (Approach 1), despite identical lesion masks and similar demographic characteristics. For the frontal MCA lesion, the spread of individual SCD scores was largest in the rolandic operculum (median [IQR]: 0.63 [0.59 – 0.67]). For the parietal MCA lesion, the greatest inter-individual variability was observed in Heschl’s gyrus (median [IQR]: 0.34 [0.28 – 0.40]). Similar patterns of variability were observed across other ROIs (Supplementary Fig. 5).

### 3.5 Correlation of SCD estimates with language outcomes in the stroke population

SCD scores derived from the model-based NeMo framework (Approach 1) and SCD scores from individual tractography (Approach 2.1) were correlated with clinical language outcomes in the stroke population. Correlations between SCD estimates and BNT, TT, and SVF were modest across ROIs and approaches (R^2^ ≤ 0.40, Supplementary Table S2). Differences in explained variance between the model-based NeMo framework (Approach 1) and individual tractography (Approach 2.1) were small across all ROIs and clinical measures (ΔR^2^ ≤ 0.08).

### 3.6 Perilesional accumulation of streamline seeds in healthy controls

Seed density maps were generated to visualize the spatial distribution of seed points used for streamline reconstruction during probabilistic tractography in healthy controls. These maps indicated increased seed density along the infarct boundary in synthetically lesioned data (Approach 2.1), whereas seed distribution in complete diffusion data (Approach 2.2) appeared spatially homogeneous across the brain. This pattern was observed in both the HCP and ADNI healthy control groups. An example case from a 68-year old male individual in the ADNI cohort is shown in Supplementary Fig. 6.

## 4 Discussion

In this study, we conducted two complementary experiments to investigate differences in structural connectivity disruption estimates. Experiment 1 compared SCD estimates from individual tractography based on subject-specific diffusion data with model-based estimates in a subacute stroke cohort. While disruption patterns were highly correlated between the two approaches, the magnitude of regional SCD differences varied across brain regions. Experiment 2 explored whether these SCD differences were influenced by the age gap between people with stroke and the younger adults in NeMo’s tractogram reference set. Synthetic lesions were applied to diffusion data from healthy young and older adults. Across most ROIs, the model-based NeMo framework (Approach 1) underestimated SCD in older adults relative to individual tractography (Approach 2.1 and 2.2), likely reflecting age-related micro- and macrostructural brain changes or differences in diffusion acquisition protocols across datasets. Differences between SCD scores derived from individual tractography performed on complete (Approach 2.2) versus synthetically lesioned data (Approach 2.1) were generally small, with the insula consistently showing larger discrepancies.

### 4.1 The effect of age on differences in SCD estimation

To investigate the impact of age in detail, identical synthetic stroke lesions were applied to diffusion data from young adults (mean ± SD: 27.8 ± 2.9 years) and older adults (mean ± SD: 75.2 ± 8.3 years). Across most ROIs, SCD was systematically underestimated by the NeMo framework (Approach 1) in older adults compared to individual tractography (Approach 2.1 and 2.2), reflecting structural characteristics not captured by the young adult reference tractogram (range: 22 – 37 years, mean ± SD: 28.7 ± 3.7 years) (Kuceyeski et al., 2013).

It is well established that stroke incidence increases with age and is most prevalent in individuals aged 65 years and older (Martin et al., 2025). WM integrity declines over the lifespan (Isaac Tseng et al., 2021), with older adults showing reduced fractional anisotropy across multiple WM tracts (Poulakis et al., 2021). These reductions are tract-specific (Sullivan and Pfefferbaum, 2006), with anterior fiber bundles being typically more affected than posterior tracts (Raffelt et al., 2017). Considerable inter-individual variability exists even among individuals of the same age (Sullivan and Pfefferbaum, 2006). Fixel-based morphometry studies have demonstrated tract-specific reductions in microscopic fiber density and macroscopic intra-axonal volume with age, consistent with neurodegenerative processes such as axonal loss and WM atrophy (Choy et al., 2020, Kennedy and Raz, 2009).

Age-related cardiovascular risk factors (CVRFs), which increase in prevalence over the lifespan, further contribute to WM deterioration (Sargurupremraj et al., 2020). Hypertension, in particular, promotes small vessel disease and the development of white matter hyperintensities (WMHs), which are visible on T2-weighted MRI and negatively affect WM integrity (Yamada et al., 2023). While the young adult cohort used to construct the model-based tractogram reference set within the NeMo framework did not have diagnosed CVRFs, the older cohort likely had a greater burden of subclinical vascular risk factors, which are not captured in the ADNI dataset. These vascular and neurodegenerative factors may contribute to the observed larger SCD differences in older adults and are not adequately accounted for in the model-based NeMo framework (Approach 1).

Beyond microstructural WM changes, age-related brain atrophy leads to reductions in GM volume (van Velsen et al., 2013), cortical thinning (Hidaka et al., 2022), and ventricular enlargement (van Niftrik et al., 2021). In stroke, these processes can be further accelerated through secondary neurodegeneration, including Wallerian degeneration (Burdette et al., 2001). Atrophy includes two major challenges for SCD estimation: First, spatial shifts in GM regions can create discrepancies between the actual anatomy and atlas-defined ROIs, causing SCD scores to misrepresent the true structural disconnection. Second, the model-based NeMo framework assumes that fibers not directly intersected by the infarct remain intact, which does not account for atrophy-related changes in connectivity.

Taken together, these findings suggest that the underestimation of SCD by the model-based NeMo framework (Approach 1) in the cohort of older adults likely results from age-related micro- and macrostructural changes, including WM degeneration, cortical atrophy, and vascular burden, which are not reflected in a young adult reference tractogram. The development of normative tractogram reference sets derived from older healthy adults would help reduce these effects, allowing for age-appropriate SCD estimation.

### 4.2 Impact of data acquisition and data processing on SCD estimation

While age-related changes are suggested to contribute to the underestimation of SCD by the model-based NeMo framework (Approach 1) in older adults, differences in diffusion acquisition protocols and processing pipelines across datasets may further modulate SCD estimates. In this study, the diffusion and structural processing pipeline was aligned as closely as possible with the pipeline used to generate the tractogram reference set in the model-based NeMo framework (Approach 1). Nevertheless, adjustments were necessary due to variations in acquisition protocols across the three datasets. Processing infarcted brains requires specialized procedures, as standard steps such as brain extraction and image registration may fail in the presence of lesions and associated distortions. In the context of this discussion, references to HCP data include both the HCP subjects used to generate the model-based tractogram reference set as well as the 30 additional HCP individuals included as a young adult comparison cohort.

Diffusion acquisition differed across datasets in multiple respects. Stroke data were acquired with a single-shell protocol (*b* = 1000 s/mm^2^), while multi-shell acquisitions were used for healthy control ADNI and HCP datasets. Multi-shell protocols are generally more robust to noise and allow for more precise estimation of fiber orientations at tissue interfaces, improving the resolution of complex fiber architectures (Jeurissen et al., 2014). The HCP protocol included a high *b*-value shell of 3000 s/mm^2^, whereas the ADNI dataset’s highest *b*-value was 2000 s/mm^2^. While higher *b*-values reduce signal-to-noise ratio (Ni et al., 2006), they increase sensitivity to microstructural features, particularly in crossing fiber regions (Ni et al., 2006).

Furthermore, stroke and ADNI scans were acquired with a single phase-encoding direction, while HCP data used a reverse phase-encoding acquisition. Bidirectional acquisitions allow more effective correction of susceptibility-induced image distortions (Schilling et al., 2020). For single-direction scans, Synb0-DisCo was used to correct for these distortions using each subject’s T1-weighted structural scan (Schilling et al., 2020). These differences in distortion correction may have affected dataset-specific diffusion quality, particularly in regions prone to susceptibility artifacts.

Other acquisition differences include the number of gradient directions (HCP: 90, ADNI: 126, NORTHSTAR: 32 or 64) and spatial resolution of diffusion data (HCP: 1.25 mm isotropic, ADNI and NORTHSTAR: 2 mm isotropic). Higher angular and spatial resolution can improve the ability to resolve crossing fibers (Sotiropoulos et al., 2013, Jones, 2004) and tractography accuracy (Ou et al., 2014).

Finally, the tractography reference space differed across cohorts. For ADNI and stroke data, tractography was performed in native diffusion space, while HCP diffusion data were registered to T1-weighted images using boundary-based registration with six degrees of freedom and then transformed to 1.25 mm T1-weighted space as part of the HCP minimal pre-processing pipeline (Glasser et al., 2013) prior to tractography. Performing tractography in native space minimizes interpolation artifacts and registration-induced distortions, which is particularly relevant in regions with high anatomical variability.

Taken together, these variations in data acquisition and processing differences could bias streamline seeding, cause false-negative and false-positive streamline reconstruction, and affect the resolution of complex fiber crossings. Consequently, they likely contribute to observed differences between SCD estimates from subject-specific tractography (Approach 2.1 and 2.2) and the model-based NeMo framework (Approach 1). Supplementary Figure 7 illustrates the tractography quality across all three datasets.

### 4.3 Regional variability in SCD estimates

Building on these dataset and age-related considerations, we next examined variability in SCD estimates across individual GM regions and subjects. While differences between approaches were generally modest, the insula consistently emerged as a region with comparatively large discrepancies. Specifically, the model-based NeMo framework (Approach 1) tended to overestimate SCD in the insula compared to individual tractography (Approach 2.1 and 2.2), particularly in older healthy adults.

The insula’s anatomical characteristics may partly explain its sensitivity to methodological differences. It is deeply embedded within the Sylvian fissure and traversed by multiple highly curved and densely packed WM pathways, including the uncinate fasciculus, arcuate fasciculus and extreme capsule fibers (Uddin et al., 2017). Its location at the interface of the frontal, temporal and parietal lobes, combined with high inter-individual variability in cortical folding, increases susceptibility to registration inaccuracies. These factors may affect streamline reconstruction and SCD estimation in this region.

Importantly, these pronounced insular differences were not observed in the comparison between the model-based NeMo framework (Approach 1) and individual tractography (Approach 2.1) within the stroke cohort, despite this cohort being of comparable age to the older healthy controls. In the stroke population, lesion size and location vary substantially across individuals, whereas in Experiment 2, identical synthetic lesion masks were applied across all subjects. The synthetic frontal lesion directly overlapped with the insula by 43%, and the parietal lesion by 7%. In subject-specific tractography, such direct overlap forces premature streamline termination or rerouting, which may disproportionately influence SCD estimates in this region. This suggests that the observed insular effects may partly reflect biases introduced by standardized synthetic lesion placement rather than region-specific biological vulnerability.

More broadly, substantial inter-individual variability was observed in SCD estimates across the 420 healthy subjects in NeMo’s tractogram reference set. Even under highly controlled conditions, with identical lesion masks and similar demographic characteristics, model-based SCD scores varied across individuals. For the insula, the IQR ranged from 0.70 to 0.74 (frontal MCA lesion) and 0.28 – 0.33 (parietal MCA lesion), while variability in other regions was greater, with IQR widths up to 0.12 in Heschl’s gyrus. These findings emphasize that normative SCD estimates are influenced by inter-individual differences in underlying structural connectivity, even when lesion and demographics are held constant.

Finally, despite these regional and inter-individual differences, correlations between SCD scores from the model-based NeMo framework (Approach 1) and individual tractography (Approach 2.1) with clinical language outcomes in the stroke cohort were similar across ROIs, with minimal differences in explained variance. This suggests that, although specific regions such as the insula may show larger methodological discrepancies, both approaches offer comparable predictive value overall.

### 4.4 The effect of seed distribution on SCD estimation

In an exploratory analysis, we examined the distribution of streamline seeds during tractography on complete (Approach 2.2) and lesioned data (Approach 2.1), with a focus on regions near lesion borders. Visual inspection of seed density maps in young and older healthy controls revealed increased seeding density at the periphery of the infarct mask relative to more distant regions in synthetically lesioned diffusion data. In probabilistic tractography, streamlines propagate along local FODs, allowing multiple potential trajectories per seed. When streamlines encounter lesioned tissue lacking diffusion signal, they are redirected along adjacent intact WM pathways, which can lead to local accumulation of streamlines near the infarct border and potentially affect SCD estimates. SIFT2 was applied to adjust streamline weights according to the underlying FOD signal (Smith et al., 2015, Hickok and Poeppel, 2007), down-weighting streamlines in regions with high local seeding density and thereby reducing perilesional accumulation.

Local seeding patterns also depend on the seeding strategy. In this study, dynamic seeding was used, whereas alternative strategies, such as GM-WM interface seeding, distribute seeds differently and could affect streamline reconstruction. Lesion-induced reductions in WM and GM volume further limit available seeding space. In this study, five million streamlines were generated per subject regardless of lesion size, the same number that was used for generation of NeMo’s tractogram reference set. Generating the same number of streamlines regardless of available seeding volume may affect SCD estimation. While SIFT2 partially compensates for these effects, future comparison studies could consider scaling the streamline count to the remaining brain volume in lesioned populations.

Taken together, these considerations suggest that local increases in seed density near infarct borders and variations in seeding strategy may contribute to differences in SCD estimates between tractography on complete (Approach 2.2) versus synthetically lesioned data (Approach 2.1).

### 4.5 Limitations

While this study provides a detailed comparison of model-based and individual tractography approaches for assessing SCD, several limitations should be considered when interpreting the results. First, the NeMo tool was used as an example of a model-based approach to assess SCD after stroke. While other model-based approaches have been developed, they were not included in this study. Nevertheless, other model-based approaches face similar constraints when estimating SCD from healthy reference tractograms, particularly in populations with age- or pathology-related structural alterations.

Second, this analysis focused on a predefined language network comprising 13 grey matter regions. While this allowed a targeted assessment of SCD in a clinically relevant network, extending the analysis to a whole-brain atlas could provide additional insights into global connectivity patterns and identify effects outside the selected ROIs.

Third, our weighting of SCD scores relied on the simplified assumption that fiber distribution and seed density are uniform within selected brain regions. Disruption scores were weighted according to the percentage of overlap between the infarct mask and a given ROI, without explicitly considering intra-regional variability in fiber density or tract complexity. This assumption was necessary because the exact number of streamlines passing through an infarcted area in lesioned tractography (Approach 2.1) is unknown compared to tractography on complete diffusion data (Approach 1 and 2.2). Nevertheless, regional refinement of the SCD weighting procedure may be required to improve accuracy.

Finally, while we explored correlations between SCD and clinical language outcomes, these analyses should be interpreted cautiously. Due to the stroke cohort’s limited sample size and the primary methodological focus of this study, these analyses were not intended to support clinical prediction. In addition, ROI-wise SCD measures are strongly interdependent due to network integration, suggesting that advanced predictive modeling, such as machine-learning approaches with larger datasets, would be needed to fully evaluate clinical relevance.

## 5 Conclusion

Individual tractography based on subject-specific diffusion data (Approach 2.1 and 2.2) and the model-based NeMo framework (Approach 1) both capture regional patterns of SCD, although the magnitude of SCD differences varies across regions and cohorts, reflecting a combination of anatomical, demographic, and technical factors. Neither approach is inherently superior. The model-based NeMo framework offers a practical and accessible option for large-scale studies or when diffusion data are unavailable, but its reliance on young adult tractograms may limit its ability to capture age- and pathology-related structural alterations. Individual tractography is more resource-intensive but may be better suited for studies focusing on age-specific or patient-specific connectivity patterns. Developing normative tractogram reference sets derived from older adults could improve the accuracy and generalizability of model-based SCD estimates.

## CRediT authorship contribution statement

**Franziska E. Hildesheim:** Writing – review & editing, Writing – original draft, Visualization, Software, Methodology, Investigation, Formal analysis, Data curation, Conceptualization. **Keith W. Jamison:** Writing – review & editing, Software, Resources, Methodology, Investigation, Data curation. **Ilana R. Leppert:** Writing – review & editing, Software. **Anna Zumbansen:** Writing – review & editing, Investigation, Data curation. **Anja Ophey:** Writing – review & editing, Investigation. **Thomas Funck:** Writing – review & editing, Software, Investigation. **Amy Kuceyeski:** Writing – review & editing, Software, Resources, Methodology, Investigation, Data curation. **Alexander Thiel:** Writing – review & editing, Supervision, Resources, Project administration, Methodology, Investigation, Funding acquisition, Formal analysis, Data curation, Conceptualization.

## Funding

This work was supported by the Canadian Institutes of Health Research (CIHR MOP#125954), the Lady Davis Institute for Medical Research (CLIPP#2014), and a Platform Support Grant (CanStim) from Brain Canada Foundation and the Canadian Partnership for Stroke Recovery. The first author F.E.H. received financial support through a Doctoral Training Scholarship from the Fonds de Recherche du Québec − Santé (FRQ-S), Government of Québec, Canada.

## Declaration of Competing Interest

The authors declare that they have no known competing financial interests or personal relationships that could have appeared to influence the work reported in this paper.

## Data Availability

Data will be made available on request.

## Glossary

AAL: Automated Anatomical Labeling
ACT: Anatomical constrained tractography
ADNI: Alzheimer’s Disease Neuroimaging Initiative
ChaCo: Change in Connectivity
CSD: Constrained spherical deconvolution
CVRF: Cardiovascular risk factor
DTI: Diffusion tensor imaging
DWI: Diffusion-weighted imaging
EMM: Estimated marginal means
FLAIR: Fluid attenuated inversion recovery
FOD: Fiber orientation distribution
GM: Grey matter
HCP: Human Connectome Project
iFOD2: Improved 2nd order integration over fiber orientation distribution
IQR: Interquartile range
LMM: Linear mixed-effects model
MCA: Middle cerebral artery
MCAFL: Frontal middle cerebral artery lesion
MCAPL: Parietal middle cerebral artery lesion
MSMT: Multi-shell multi-tissue
MNI: Montréal Neurological Institute
MRI: Magnetic resonance imaging
NeMo: Network Modification
NIHSS: National Institutes of Health Stroke Scale
NORTHSTAR: Non-Invasive Repeated Therapeutic Stimulation for Aphasia Recovery
ROI: Region of interest
SCD: Structural connectivity disruption
SIFT: Spherical-deconvolution Informed Filtering of Tractograms
SS3T: Single-shell 3-tissue
Synb0-DisCo: Synthesized b0 for diffusion distortion correction
WM: White matter
BNT: Boston Naming Test
CSF: Cerebrospinal Fluid
TT: Token Test
SVF: Semantic Verbal Fluency Test
T: Tesla
TE: echo time
TI: Inversion time
TR: repetition time
IFG: Inferior frontal gyrus
STG: Superior temporal gyrus
WMH: White matter hyperintensities
HCP-YA: Human Connectome Project-Young Adult

## References

[b0005] Martin S.S., Aday A.W., Allen N.B., Almarzooq Z.I., Anderson C.A.M., Arora P. (2025). 2025 Heart Disease and Stroke Statistics: a Report of US and Global Data from the American Heart Association. Circulation.

[b0010] Gallucci L., Sperber C., Guggisberg A.G., Kaller C.P., Heldner M.R., Monsch A.U. (2024). Post-stroke cognitive impairment remains highly prevalent and disabling despite state-of-the-art stroke treatment. Int. J. Stroke.

[b0015] Einstad M.S., Saltvedt I., Lydersen S., Ursin M.H., Munthe-Kaas R., Ihle-Hansen H. (2021). Associations between post-stroke motor and cognitive function: a cross-sectional study. BMC Geriatr..

[b0020] Cassidy J.M., Cramer S.C. (2017). Spontaneous and Therapeutic-Induced Mechanisms of Functional Recovery after Stroke. Transl. Stroke Res..

[b0025] Ramsey L.E., Siegel J.S., Lang C.E., Strube M., Shulman G.L., Corbetta M. (2017). Behavioural clusters and predictors of performance during recovery from stroke. Nat. Hum Behav..

[b0030] Osa Garcia A., Brambati S.M., Brisebois A., Desilets-Barnabe M., Houze B., Bedetti C. (2020). Predicting Early Post-stroke Aphasia Outcome from initial Aphasia Severity. Front. Neurol..

[b0035] Johnson L., Nemati S., Bonilha L., Rorden C., Busby N., Basilakos A. (2022). Predictors beyond the lesion: Health and demographic factors associated with aphasia severity. Cortex.

[b0040] Kim T.H., Vemuganti R. (2015). Effect of sex and age interactions on functional outcome after stroke. CNS Neurosci. Ther..

[b0045] Rexrode K.M., Madsen T.E., Yu A.Y.X., Carcel C., Lichtman J.H., Miller E.C. (2022). The Impact of sex and Gender on Stroke. Circ. Res..

[b0050] Hildesheim F.E., Ophey A., Zumbansen A., Funck T., Schuster T., Jamison K.W. (2024). Predicting Language Function Post-Stroke: a Model-based Structural Connectivity Approach. Neurorehabil. Neural Repair.

[b0055] Aam S., Gynnild M.N., Munthe-Kaas R., Saltvedt I., Lydersen S., Knapskog A.B. (2021). The Impact of Vascular Risk Factors on Post-stroke Cognitive Impairment: the Nor-COAST Study. Front. Neurol..

[b0060] Roth R., Busby N., Wilmskoetter J., Schwen Blackett D., Gleichgerrcht E., Johnson L. (2023). Diabetes, brain health, and treatment gains in post-stroke aphasia. Cereb. Cortex.

[b0065] Menichelli A., Furlanis G., Sartori A., Ridolfi M., Naccarato M., Caruso P. (2019). Thrombolysis' benefits on early post-stroke language recovery in aphasia patients. J. Clin. Neurosci..

[b0070] Yvette S. Crijnen, Femke Nouwens, Lonneke M.L. de Lau, Evy G. Visch-Brink, Mieke W.M.E. van de Sandt-Koenderman, Olvert A. Berkhemer, et al. Early effect of intra-arterial treatment in ischemic stroke on aphasia recovery in MR CLEAN. Neurology. 2016;86.

[b0075] Kristinsson S., Fridriksson J. (2022). Genetics in aphasia recovery. Handb. Clin. Neurol..

[b0080] Balkaya M., Cho S. (2019). Genetics of stroke recovery: BDNF val66met polymorphism in stroke recovery and its interaction with aging. Neurobiol. Dis..

[b0085] Hillis A.E., Beh Y.Y., Sebastian R., Breining B., Tippett D.C., Wright A. (2018). Predicting recovery in acute poststroke aphasia. Ann. Neurol..

[b0090] Feng W., Wang J., Chhatbar P.Y., Doughty C., Landsittel D., Lioutas V.A. (2015). Corticospinal tract lesion load: an imaging biomarker for stroke motor outcomes. Ann. Neurol..

[b0095] Salvalaggio A, De Filippo De Grazia M, Zorzi M, Thiebaut de Schotten M, Corbetta M. Post-stroke deficit prediction from lesion and indirect structural and functional disconnection. Brain. 2020;143(7):2173-88. 10.1093/brain/awaa156 PMC7363494 32572442

[b0100] Bowren M., Bruss J., Manzel K., Edwards D., Liu C., Corbetta M. (2022). Post-stroke outcomes predicted from multivariate lesion-behaviour and lesion network mapping. Brain.

[b0105] Thiel A., Zumbansen A. (2016). The pathophysiology of post-stroke aphasia: a network approach. Restor. Neurol. Neurosci..

[b0110] Dmytriw A.A., Sawlani V., Shankar J. (2017). Diffusion-Weighted Imaging of the Brain: beyond Stroke. Can. Assoc. Radiol. J..

[b0115] Tae W.S., Ham B.J., Pyun S.B., Kang S.H., Kim B.J. (2018). Current Clinical applications of Diffusion-Tensor Imaging in Neurological Disorders. J Clin Neurol..

[b0120] Jeurissen B., Leemans A., Tournier J.D., Jones D.K., Sijbers J. (2013). Investigating the prevalence of complex fiber configurations in white matter tissue with diffusion magnetic resonance imaging. Hum. Brain Mapp..

[b0125] Auriat A.M., Borich M.R., Snow N.J., Wadden K.P., Boyd L.A. (2015). Comparing a diffusion tensor and non-tensor approach to white matter fiber tractography in chronic stroke. NeuroImage: Clinical..

[b0130] Jeurissen B., Tournier J.D., Dhollander T., Connelly A., Sijbers J. (2014). Multi-tissue constrained spherical deconvolution for improved analysis of multi-shell diffusion MRI data. Neuroimage.

[b0135] Behrens T.E., Berg H.J., Jbabdi S., Rushworth M.F., Woolrich M.W. (2007). Probabilistic diffusion tractography with multiple fibre orientations: what can we gain?. Neuroimage.

[b0140] Jeurissen B., Leemans A., Jones D.K., Tournier J.D., Sijbers J. (2011). Probabilistic fiber tracking using the residual bootstrap with constrained spherical deconvolution. Hum. Brain Mapp..

[b0145] Kuceyeski A., Maruta J., Relkin N., Raj A. (2013). The Network Modification (NeMo) Tool: elucidating the effect of white matter integrity changes on cortical and subcortical structural connectivity. Brain Connect..

[b0150] Griffis J.C., Metcalf N.V., Corbetta M., Shulman G.L. (2021). Lesion Quantification Toolkit: a MATLAB software tool for estimating grey matter damage and white matter disconnections in patients with focal brain lesions. Neuroimage Clin..

[b0155] Foulon C., Cerliani L., Kinkingnehun S., Levy R., Rosso C., Urbanski M. (2018). Advanced lesion symptom mapping analyses and implementation as BCBtoolkit. GigaScience.

[b0160] Kuceyeski A., Kamel H., Navi B.B., Raj A., Iadecola C. (2014). Predicting future brain tissue loss from white matter connectivity disruption in ischemic stroke. Stroke.

[b0165] Schaechter J.D., Kim M., Hightower B.G., Ragas T., Loggia M.L. (2023). Disruptions in Structural and Functional Connectivity Relate to Poststroke Fatigue. Brain Connect..

[b0170] Kuceyeski A.F., Vargas W., Dayan M., Monohan E., Blackwell C., Raj A. (2015). Modeling the relationship among gray matter atrophy, abnormalities in connecting white matter, and cognitive performance in early multiple sclerosis. AJNR Am. J. Neuroradiol..

[b0175] Tozlu C., Jamison K., Gu Z., Gauthier S.A., Kuceyeski A. (2021). Estimated connectivity networks outperform observed connectivity networks when classifying people with multiple sclerosis into disability groups. Neuroimage Clin..

[b0180] Thiel A., Black S.E., Rochon E.A., Lanthier S., Hartmann A., Chen J.L. (2015). Non-invasive repeated therapeutic stimulation for aphasia recovery: a multilingual, multicenter aphasia trial. J. Stroke Cerebrovasc. Dis..

[b0185] Van Essen D.C., Smith S.M., Barch D.M., Behrens T.E., Yacoub E., Ugurbil K. (2013). The WU-Minn Human Connectome Project: an overview. Neuroimage.

[b0190] Mueller S.G., Weiner M.W., Thal L.J., Petersen R.C., Jack C., Jagust W. (2005). The Alzheimer's disease neuroimaging initiative. Neuroimaging Clin. N. Am..

[b0195] Brunelli S., Giannella E., Bizzaglia M., De Angelis D., Sancesario G.M. (2023). Secondary neurodegeneration following Stroke: what can blood biomarkers tell us?. Front. Neurol..

[b0200] Goodman G.W., Do T.H., Tan C., Ritzel R.M. (2023). Drivers of Chronic Pathology following Ischemic Stroke: a Descriptive Review. Cell. Mol. Neurobiol..

[b0205] Tombaugh T.N., Hubley A.M. (1997). The 60-item Boston Naming Test: norms for cognitively intact adults aged 25 to 88 years. J. Clin. Exp. Neuropsychol..

[b0210] De Renzi E., Faglioni P. (1978). Normative Data and Screening Power of a Shortened Version of the Token Test. Cortex.

[b0215] Tombaugh T.N. (1999). Normative Data Stratified by Age and Education for two measures of Verbal Fluency: FAS and Animal Naming. Arch. Clin. Neuropsychol..

[b0220] Glasser M.F., Sotiropoulos S.N., Wilson J.A., Coalson T.S., Fischl B., Andersson J.L. (2013). The minimal preprocessing pipelines for the Human Connectome Project. Neuroimage.

[b0225] Liu C.F., Hsu J., Xu X., Kim G., Sheppard S.M., Meier E.L. (2023). Digital 3D Brain MRI Arterial Territories Atlas. Sci. Data.

[b0230] Tzourio-Mazoyer N., Landeau B., Papathanassiou D., Crivello F., Etard O., Delcroix N. (2002). Automated anatomical labeling of activations in SPM using a macroscopic anatomical parcellation of the MNI MRI single-subject brain. Neuroimage.

[b0235] Schilling K.G., Blaber J., Huo Y., Newton A., Hansen C., Nath V. (2019). Synthesized b0 for diffusion distortion correction (Synb0-DisCo). Magn. Reson. Imaging.

[b0240] Schilling K.G., Blaber J., Hansen C., Cai L., Rogers B., Anderson A.W. (2020). Distortion correction of diffusion weighted MRI without reverse phase-encoding scans or field-maps. PLoS One.

[b0245] Andersson J.L.R., Sotiropoulos S.N. (2016). An integrated approach to correction for off-resonance effects and subject movement in diffusion MR imaging. Neuroimage.

[b0250] Jenkinson M., Beckmann C.F., Behrens T.E., Woolrich M.W., Smith S.M. (2012). Fsl. Neuroimage..

[b0255] Fischl B. (2012). FreeSurfer. Neuroimage..

[b0260] Jenkinson M., Bannister P., Brady M., Smith S. (2002). Improved Optimization for the Robust and Accurate Linear Registration and Motion Correction of Brain Images. Neuroimage.

[b0265] Avants B.B., Epstein C.L., Grossman M., Gee J.C. (2008). Symmetric Diffeomorphic image Registration with CrossCorrelation: evaluating Automated labeling of elderly and Neurodegenerative Brain. Med. Image Anal..

[b0270] Smith R.E., Tournier J.D., Calamante F., Connelly A. (2012). Anatomically-constrained tractography: improved diffusion MRI streamlines tractography through effective use of anatomical information. Neuroimage.

[b0275] Dhollander T., Raffelt D., Connelly A. (2016). SMRM Workshop on Breaking the Barriers of Diffusion MRI.

[b0280] Dhollander T., Connell A. (2016). A novel iterative approach to reap the benefits of multi-tissue CSD from just single-shell (+b=0) diffusion MRI data. Proc Intl Soc Mag Reson Med..

[b0285] Tournier J.D., Calamante F., Gadian D.G., Connelly A. (2004). Direct estimation of the fiber orientation density function from diffusion-weighted MRI data using spherical deconvolution. Neuroimage.

[b0290] Tournier J.D., Calamante F., Connelly A. (2010). Improved probabilistic streamlines tractography by 2nd order integration over fibre orientation distributions. Proc Intl Soc Mag Reson Med..

[b0295] Smith R.E., Tournier J.D., Calamante F., Connelly A. (2015). SIFT2: Enabling dense quantitative assessment of brain white matter connectivity using streamlines tractography. Neuroimage.

[b0300] Smith R.E., Tournier J.D., Calamante F., Connelly A. (2015). The effects of SIFT on the reproducibility and biological accuracy of the structural connectome. Neuroimage.

[b0305] Hickok G., Poeppel D. (2007). The cortical organization of speech processing. Nat. Rev. Neurosci..

[b0310] Price C.J. (2012). A review and synthesis of the first 20 years of PET and fMRI studies of heard speech, spoken language and reading. Neuroimage.

[b0315] Fridriksson J., den Ouden D.B., Hillis A.E., Hickok G., Rorden C., Basilakos A. (2018). Anatomy of aphasia revisited. Brain.

[b0320] Fujii M., Maesawa S., Ishiai S., Iwami K., Futamura M., Saito K. (2016). Neural Basis of Language: an Overview of an Evolving Model. Neurol. Med. Chir. (Tokyo).

[b0325] Ardila A., Bernal B., Rosselli M. (2016). How Localized are Language Brain areas? a Review of Brodmann areas Involvement in Oral Language. Arch. Clin. Neuropsychol..

[b0330] Bennett I.J., Madden D.J. (2014). Disconnected aging: cerebral white matter integrity and age-related differences in cognition. Neuroscience.

[b0335] Isaac Tseng W.Y., Hsu Y.C., Chen C.L., Kang Y.J., Kao T.W., Chen P.Y. (2021). Microstructural differences in white matter tracts across middle to late adulthood: a diffusion MRI study on 7167 UK Biobank participants. Neurobiol. Aging.

[b0340] Poulakis K., Reid R.I., Przybelski S.A., Knopman D.S., Graff-Radford J., Lowe V.J. (2021). Longitudinal deterioration of white-matter integrity: heterogeneity in the ageing population. Brain Commun..

[b0345] Sullivan E.V., Pfefferbaum A. (2006). Diffusion tensor imaging and aging. Neurosci. Biobehav. Rev..

[b0350] Raffelt D.A., Tournier J.D., Smith R.E., Vaughan D.N., Jackson G., Ridgway G.R. (2017). Investigating white matter fibre density and morphology using fixel-based analysis. Neuroimage.

[b0355] Choy S.W., Bagarinao E., Watanabe H., Ho E.T.W., Maesawa S., Mori D. (2020). Changes in white matter fiber density and morphology across the adult lifespan: a cross-sectional fixel-based analysis. Hum. Brain Mapp..

[b0360] Kennedy K.M., Raz N. (2009). Pattern of normal age-related regional differences in white matter microstructure is modified by vascular risk. Brain Res..

[b0365] Sargurupremraj M., Suzuki H., Jian X., Sarnowski C., Evans T.E., Bis J.C. (2020). Cerebral small vessel disease genomics and its implications across the lifespan. Nat. Commun..

[b0370] Yamada S., Otani T., Ii S., Kawano H., Nozaki K., Wada S. (2023). Aging-related volume changes in the brain and cerebrospinal fluid using artificial intelligence-automated segmentation. Eur. Radiol..

[b0375] van Velsen E.F., Vernooij M.W., Vrooman H.A., van der Lugt; A., Breteler M.M., Hofman A. (2013). Brain cortical thickness in the general elderly population: the Rotterdam Scan Study. Neurosci. Lett..

[b0380] Hidaka Y., Hashimoto M., Suehiro T., Fukuhara R., Ishikawa T., Tsunoda N. (2022). Impact of age on the cerebrospinal fluid spaces: high-convexity and medial subarachnoid spaces decrease with age. Fluids Barriers CNS.

[b0385] van Niftrik C.H.B., Sebok M., Muscas G., Wegener S., Luft A.R., Stippich C. (2021). Investigating the Association of Wallerian Degeneration and Diaschisis after Ischemic Stroke with BOLD Cerebrovascular Reactivity. Front. Physiol..

[b0390] Burdette J.H., Durden D.D., Elster A.D., Yen Y.F. (2001). High b-Value Diffusion-Weighted MRI of Normal Brain. J. Comput. Assist. Tomogr..

[b0395] Ni H, Kavcic V, Zhu T, Ekholm S, J. Z. Effects of number of diffusion gradient directions on derived diffusion tensor imaging indices in human brain. American Journal of Neuroradiology. 2006;27(8):1776-81. PMC8139764 16971635

[b0400] Sotiropoulos S.N., Jbabdi S., Xu J., Andersson J.L., Moeller S., Auerbach E.J. (2013). Advances in diffusion MRI acquisition and processing in the Human Connectome Project. Neuroimage.

[b0405] Jones D.K. (2004). The effect of gradient sampling schemes on measures derived from diffusion tensor MRI: a Monte Carlo study. Magn. Reson. Med..

[b0410] Ou Y., Akbari H., Bilello M., Da X., Davatzikos C. (2014). Comparative evaluation of registration algorithms in different brain databases with varying difficulty: results and insights. IEEE Trans. Med. Imaging.

[b0415] Uddin L.Q., Nomi J.S., Hebert-Seropian B., Ghaziri J., Boucher O. (2017). Structure and Function of the Human Insula. J. Clin. Neurophysiol..

