# Supplementary material for: Evaluating structural connectivity disruption after stroke: individual tractography or the use of a model-based approach?
Source: Neuroimage Clin. 2026 Feb 18;49:103967. doi: 10.1016/j.nicl.2026.103967 (PMC13080591; doi:10.1016/j.nicl.2026.103967)
Supplement: Supplementary Data 1 [file mmc1.docx]

**Supplementary Figures**

**Supplementary Figure 1**: Data processing pipeline.

Boxes with a white background indicate steps applied across all three cohorts. Colored boxes denote cohort-specific steps: Orange for NORTHSTAR & ADNI, yellow for NORTHSTAR only, blue for ADNI only, and lavender for ADNI & HCP. Boxes with a solid outline represent steps applied in Approach 2.1 (tractography on naturally or synthetically lesioned diffusion data) and Approach 2.2 (tractography on complete diffusion data), while boxes with a dashed outline indicate steps applied only in Approach 2.1 (tractography on naturally or synthetically lesioned diffusion data). Software tools used: FSL, Freesurfer, ANTs, and MRtrix3. NORTHSTAR: Non-Invasive Repeated Therapeutic Stimulation for Aphasia Recovery, HCP: Human Connectome Project, ADNI: Alzheimer’s Disease Neuroimaging Initiative, DWI: diffusion-weighted imaging, CSD: constrained spherical deconvolution, ACT: anatomical constrained tractography, Synb0-DisCo: synthesized b0 for diffusion distortion correction, SyN: symmetric normalization, AAL: Automated Anatomical Labeling, WM: white matter, GM: grey matter, ROI: region of interest, MNI: Montréal Neurological Institute, DOF: degrees of freedom.

**Supplementary Figure 2**: Correlation between structural connectivity disruption scores estimated with the model-based Network Modification tool versus individual tractography in people with stroke (Experiment 1).

Structural connectivity disruption (SCD) scores derived from the model-based Network Modification framework (Approach 1) are plotted against absolute SCD scores derived from individual tractography (Approach 2.1) for 13 grey matter regions, shown separately for each participant. A regression line with 95% confidence interval is overlaid. NeMo: Network Modification tool, ChaCo: Change in Connectivity, SCD: structural connectivity disruption.

**Supplementary Figure 3:** Differences in structural connectivity disruption scores estimated with individual tractography on complete versus lesioned diffusion data in healthy adults (Experiment 2, Approach 2.2 vs. Approach 2.1).

Glass brain visualizations show median differences in structural connectivity disruption (SCD) scores across 13 grey matter regions from the AAL116 atlas in young and older adults. Blue indicates underestimation by individual tractography on complete diffusion data (Approach 2.2) relative to synthetically lesioned data (Approach 2.1), whereas red indicates overestimation. Tables: Results from rank-based linear mixed effects models, with cohort and regions of interest (ROI) as fixed effects and subject as a random intercept. Estimated marginal means (EMMs) of ranked SCD differences were calculated for each cohort and ROI, with pairwise contrasts between young and older adults. Statistical significance was defined as *p* ≤ 0.05 (*). HCP: Human Connectome Project, ADNI: Alzheimer’s Disease Neuroimaging Initiative, MCA: middle cerebral artery, SCD: structural connectivity disruption, IFG: inferior frontal gyrus, AAL: Automated Anatomic Labeling, ROI: region of interest, EMM: estimated marginal means, IQR: interquartile range, L: left, R: right.

**Supplementary Figure 4:** Differences in structural connectivity disruption scores estimated with the model-based Network Modification tool versus individual tractography on complete diffusion data of healthy adults (Experiment 2, Approach 1 vs. Approach 2.2).

Glass brain visualizations show median differences in structural connectivity disruption (SCD) scores across 13 grey matter regions from the AAL116 atlas in young and older adults. Blue indicates underestimation of the model-based NeMo framework (Approach 1) relative to individual tractography on complete diffusion data (Approach 2.2), whereas red indicates overestimation. Tables: Results from rank-based linear mixed effects models, with cohort and regions of interest (ROIs) as fixed effects and subject as a random intercept. Estimated marginal means (EMMs) of ranked SCD differences were calculated for each cohort and ROI, with pairwise contrasts between young and older adults. Statistical significance was defined as *p* ≤ 0.05 (*). HCP: Human Connectome Project, ADNI: Alzheimer’s Disease Neuroimaging Initiative, MCA: middle cerebral artery, SCD: structural connectivity disruption, IFG: inferior frontal gyrus, AAL: Automated Anatomic Labeling, ROI: region of interest, EMM: estimated marginal means, IQR: interquartile range, L: left, R: right.

**Supplementary Figure 5:** Variability of structural connectivity disruption scores across 420 individual tractograms in the reference set used by the model-based Network Modification tool (Approach 1).

The distribution of structural connectivity disruption scores, termed Change in Connectivity (ChaCo) scores, within the NeMo framework (Approach 1), is shown for 13 grey matter regions of interest across the 420 individual tractograms included in the NeMo reference set, displayed for the synthetic frontal and parietal MCA lesion. Boxes indicate the interquartile range, with the horizontal line representing the median. Whiskers extend to the most extreme values within 1.5 times the interquartile range and values beyond this range are plotted as outliers (circles). NeMo: Network Modification tool, ChaCo: Change in Connectivity, MCA: middle cerebral artery, IFG: inferior frontal gyrus.

**Supplementary Figure 6:** Seed distribution in tractography performed on complete versus synthetically lesioned diffusion data in a representative healthy adult.

Seed density maps show the spatial distribution of seed points used for probabilistic tractography with dynamic seeding. Top panel: Seed distribution from tractography performed on complete diffusion data (Approach 2.2). Bottom panel: Seed distribution from tractography performed on lesioned diffusion data (Approach 2.1). Brighter colors indicate higher seed density. The subject’s T1-weighted structural image, registered to native diffusion space, is shown in the background. This example is from a 68-year-old male healthy adult from the ADNI cohort. ADNI: Alzheimer’s Disease Neuroimaging Initiative, L: left, R: right.

**Supplementary Figure 7**: Exemplar tractograms illustrating data quality across datasets.

Representative tractograms are shown for each of the three datasets. Left: example tractogram from the NORTHSTAR stroke cohort. Middle: example tractogram from synthetically lesioned HCP data (frontal MCA lesion). Right: example tractogram from synthetically lesioned ADNI data (parietal MCA lesion). The top row shows axial views, and the bottom row shows coronal views. Images were generated using mrview (MRtrix3). NORTHSTAR: Non-Invasive Repeated Therapeutic Stimulation for Aphasia Recovery, HCP: Human Connectome Project, ADNI: Alzheimer’s Disease Neuroimaging Initiative, MCA: middle cerebral artery, L: left, R: right.

**Supplementary Tables**

**Supplementary Table 1**: Selection of regions of interest involved in speech and language processing and production.

The functional characterization of language-related regions of interest is based on established neurocognitive models and comprehensive reviews of language processing. IFG: inferior frontal gyrus.

**Supplementary Table 2**: Associations between structural connectivity disruption scores and language outcomes in the stroke population.

Structural connectivity disruption (SCD) scores derived from the model-based NeMo framework (Approach 1) and from individual tractography (Approach 2.1) were correlated with language outcomes assessed six weeks after baseline, including a) Boston Naming Test, b) Token Test, and c) Semantic Verbal Fluency Test. R² values indicate the proportion of variance in each language outcome explained by SCD estimates for each ROI, reported separately for the two approaches. Differences in explained variance (ΔR²) between the NeMo framework (R^2^ NeMo) and individual tractography (R^2^ DWI) are also provided. SCD: structural connectivity disruption, NeMo: Network Modification tool, DWI: diffusion-weighted imaging, FU: follow-up, ROI: region of interest, IFG: inferior frontal gyrus.
