# Supplementary material for: Evaluating structural connectivity disruption after stroke: individual tractography or the use of a model-based approach?
Source: Neuroimage Clin. 2026 Feb 18;49:103967. doi: 10.1016/j.nicl.2026.103967 (PMC13080591; doi:10.1016/j.nicl.2026.103967)
Supplement: Supplementary Data 2 [file mmc2.docx]

**Supplementary Table 1**

| **Region of Interest** | **Primary Role in Language Function** |
| --- | --- |
| Precentral gyrus | Motor execution of speech; control of larynx, tongue, lips, and jaw |
| IFG pars opercularis | Speech production and articulatory planning; grammatical and syntactic processing |
| IFG pars triangularis | Controlled semantic retrieval; selection of competing word meanings; semantic aspects of language production |
| Rolandic operculum | Speech motor control; coordination of articulatory movements; sensorimotor integration during speech production |
| Insula | Speech articulation and motor planning; coordination of complex speech movements |
| Inferior parietal gyrus | Semantic processing; phonological-semantic integration; language-related working memory and attention |
| Supramarginal gyrus | Phonological processing; mapping of sound to articulation; speech repetition |
| Angular gyrus | Semantic integration across sentences and discourse; linking language with memory, attention, and conceptual processing |
| Heschl’s gyrus | Initial cortical processing of auditory input; extraction of basic acoustic features from incoming speech |
| Superior temporal gyrus | Early auditory and phonological processing; integration of phonemes into syllables; transmission of phonological representations to motor planning areas for speech production |
| Superior temporal pole | Semantic memory for words and concepts; integration of semantic information across modalities |
| Middle temporal gyrus | Lexical-semantic processing; mapping phonological representations to meaning; semantic integration |
| Middle temporal pole | Semantic associations; naming and lexical retrieval |
