# Supplementary material for: Evaluating structural connectivity disruption after stroke: individual tractography or the use of a model-based approach?
Source: Neuroimage Clin. 2026 Feb 18;49:103967. doi: 10.1016/j.nicl.2026.103967 (PMC13080591; doi:10.1016/j.nicl.2026.103967)
Supplement: Supplementary Data 3 [file mmc3.docx]

**Supplementary Table 2**

1. **Boston Naming Test**

| **ROI** | **R^2^ NeMo** | **R^2^ DWI** | Δ**R^2^** |
| --- | --- | --- | --- |
| Precentral gyrus | 0.27 | 0.25 | 0.02 |
| IFG pars opercularis | 0.29 | 0.31 | -0.02 |
| IFG pars triangularis | 0.27 | 0.28 | -0.01 |
| Rolandic operculum | 0.32 | 0.40 | -0.08 |
| Insula | 0.28 | 0.29 | -0.01 |
| Inferior parietal gyrus | 0.10 | 0.09 | 0.01 |
| Supramarginal gyrus | 0.26 | 0.20 | 0.06 |
| Angular gyrus | 0.06 | 0.05 | 0.01 |
| Heschl’s gyrus | 0.20 | 0.27 | -0.07 |
| Superior temporal gyrus | 0.24 | 0.27 | -0.03 |
| Superior temporal pole | 0.24 | 0.22 | 0.02 |
| Middle temporal gyrus | 0.19 | 0.19 | 0.00 |
| Middle temporal pole | 0.13 | 0.11 | 0.02 |

1. **Token Test**

| **ROI** | **R^2^ NeMo** | **R^2^ DWI** | **ΔR^2^** |
| --- | --- | --- | --- |
| Precentral gyrus | 0.11 | 0.12 | -0.01 |
| IFG pars opercularis | 0.18 | 0.19 | -0.01 |
| IFG pars triangularis | 0.21 | 0.20 | 0.01 |
| Rolandic operculum | 0.18 | 0.24 | -0.06 |
| Insula | 0.18 | 0.18 | 0.00 |
| Inferior parietal gyrus | 0.04 | 0.05 | -0.01 |
| Supramarginal gyrus | 0.17 | 0.14 | 0.03 |
| Angular gyrus | 0.06 | 0.06 | 0.00 |
| Heschl’s gyrus | 0.17 | 0.25 | -0.08 |
| Superior temporal gyrus | 0.25 | 0.28 | -0.03 |
| Superior temporal pole | 0.13 | 0.13 | 0.00 |
| Middle temporal gyrus | 0.21 | 0.21 | 0.00 |
| Middle temporal pole | 0.02 | 0.05 | -0.03 |

1. **Semantic Verbal Fluency Test**

| **ROI** | **R^2^ NeMo** | **R^2^ DWI** | Δ**R^2^** |
| --- | --- | --- | --- |
| Precentral gyrus | 0.31 | 0.34 | -0.03 |
| IFG pars opercularis | 0.31 | 0.33 | -0.02 |
| IFG pars triangularis | 0.29 | 0.30 | -0.01 |
| Rolandic operculum | 0.29 | 0.37 | -0.08 |
| Insula | 0.26 | 0.25 | 0.01 |
| Inferior parietal gyrus | 0.09 | 0.09 | 0.00 |
| Supramarginal gyrus | 0.22 | 0.18 | 0.04 |
| Angular gyrus | 0.03 | 0.03 | 0.00 |
| Heschl’s gyrus | 0.14 | 0.20 | -0.06 |
| Superior temporal gyrus | 0.16 | 0.19 | -0.03 |
| Superior temporal pole | 0.14 | 0.10 | 0.04 |
| Middle temporal gyrus | 0.12 | 0.12 | 0.00 |
| Middle temporal pole | 0.06 | 0.04 | 0.02 |
