# Supplementary figures and images for: Evaluating structural connectivity disruption after stroke: individual tractography or the use of a model-based approach?
Source: Neuroimage Clin. 2026 Feb 18;49:103967. doi: 10.1016/j.nicl.2026.103967 (PMC13080591; doi:10.1016/j.nicl.2026.103967)

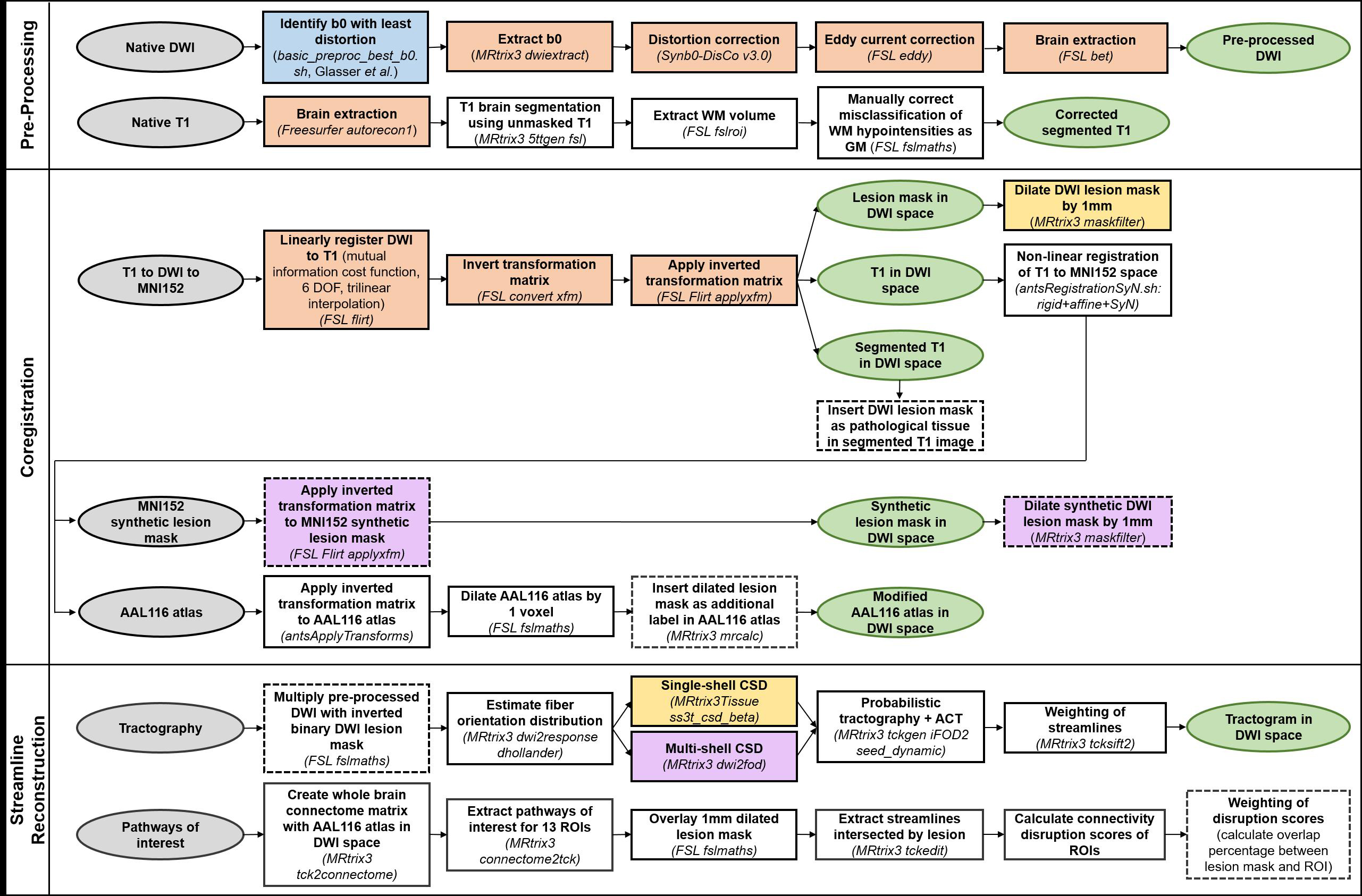

Supplement: Supplementary Figure 4 [file mmc4.jpg]

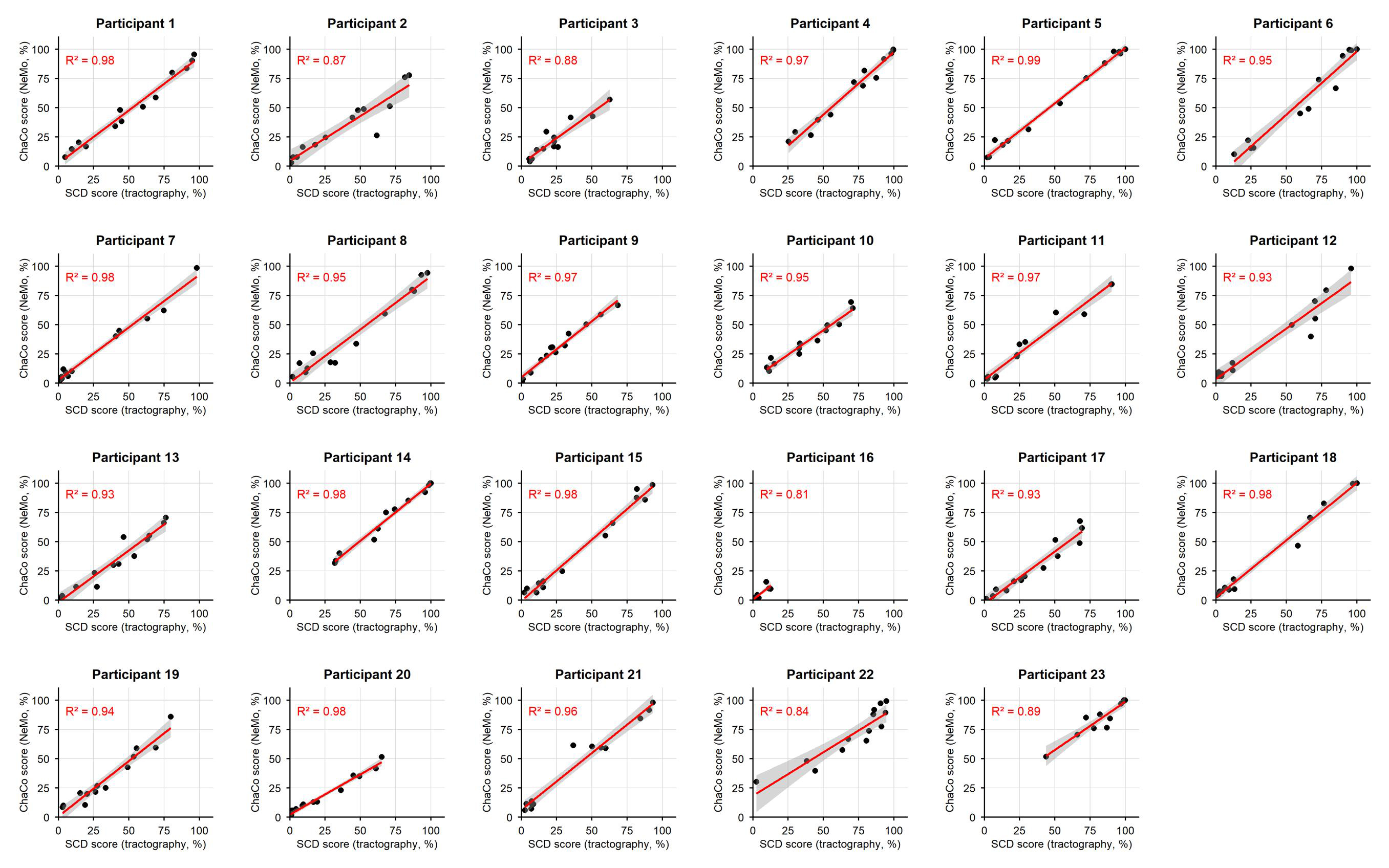

Supplement: Supplementary Figure 5 [file mmc5.jpg]

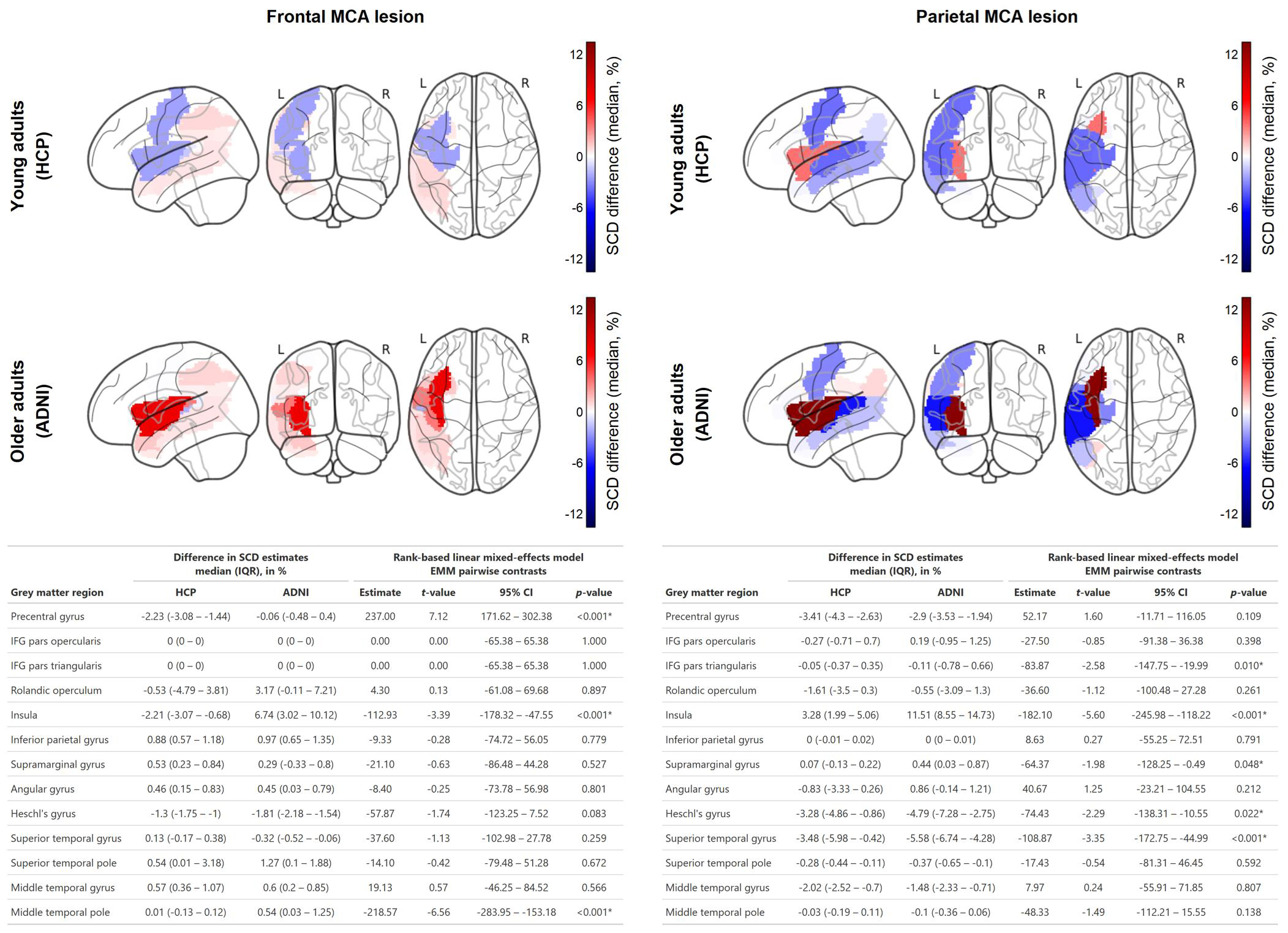

Supplement: Supplementary Figure 6 [file mmc6.jpg]

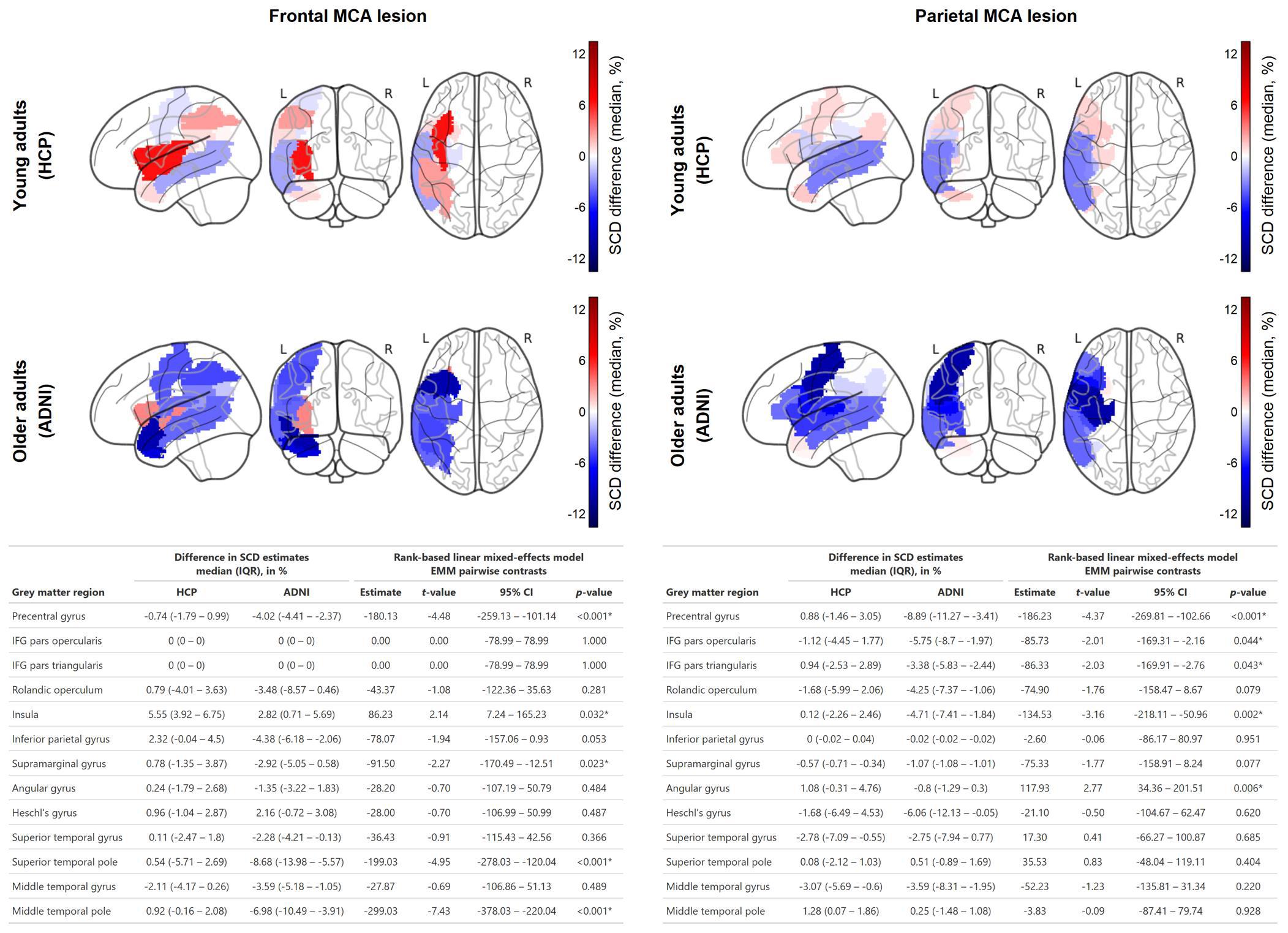

Supplement: Supplementary Figure 7 [file mmc7.jpg]

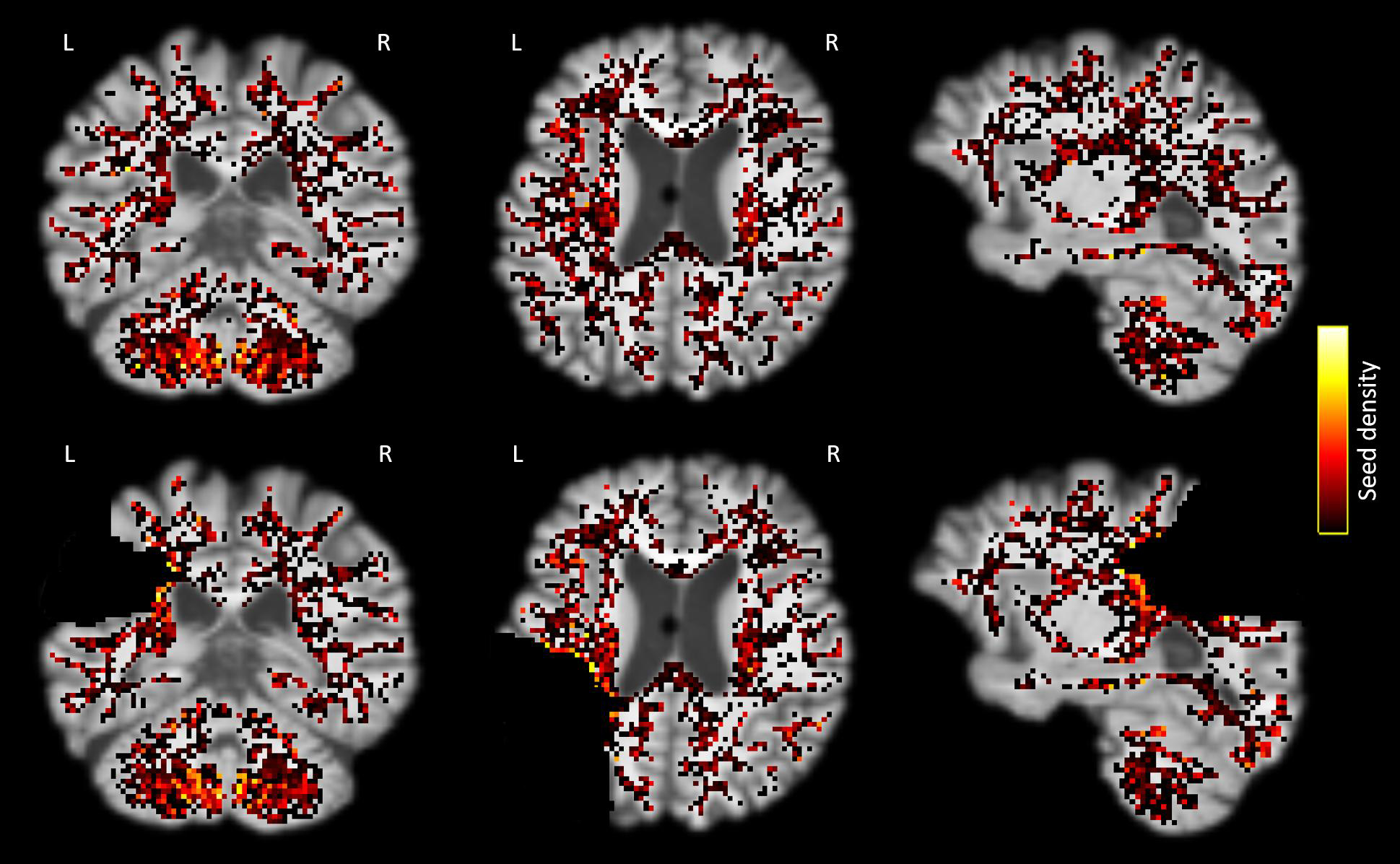

Supplement: Supplementary Figure 8 [file mmc8.jpg]

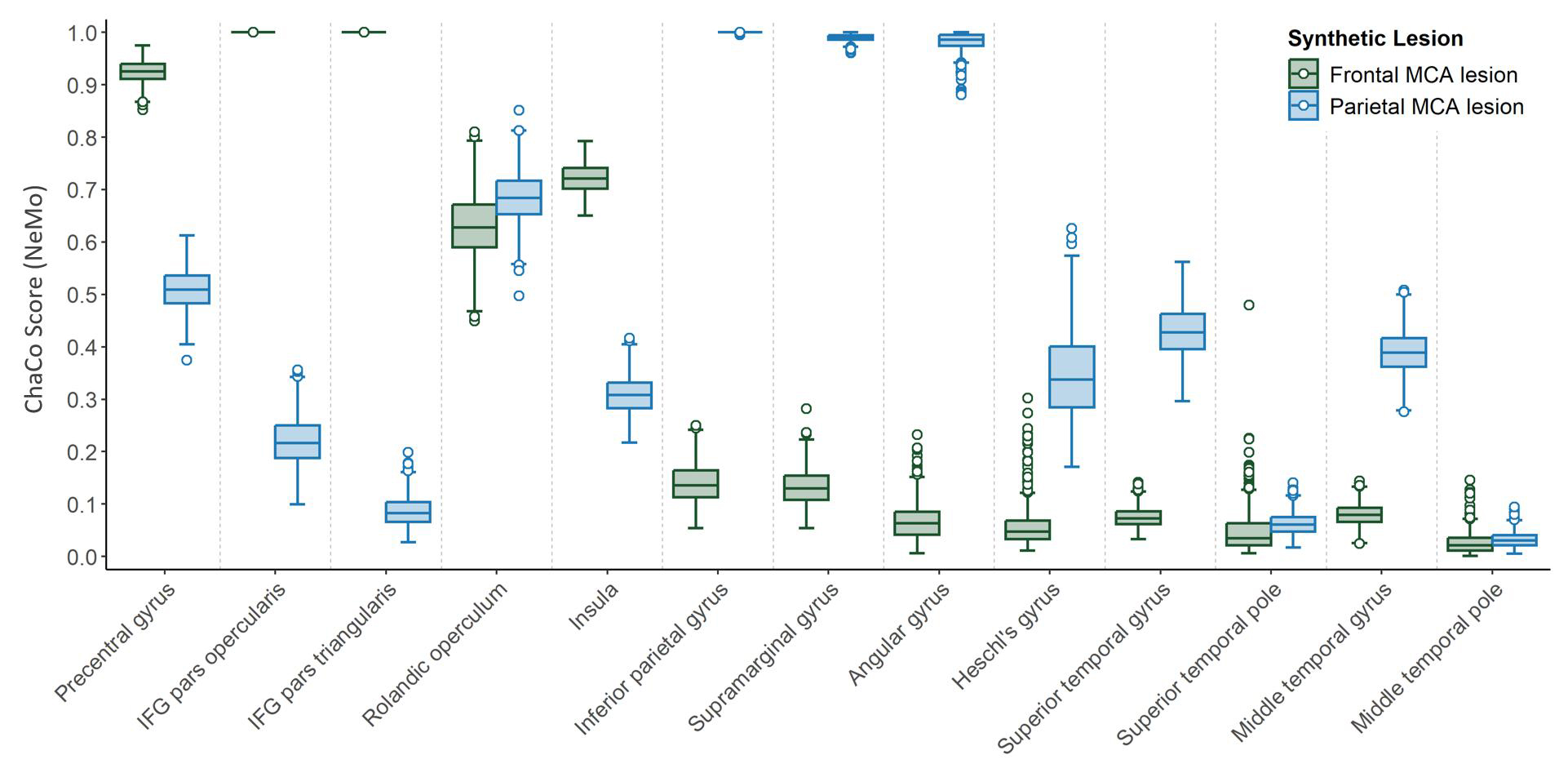

Supplement: Supplementary Figure 9 [file mmc9.jpg]
